# The Nucleosome Acidic Patch Regulates the H2B K123 Monoubiquitylation Cascade and Transcription Elongation in *Saccharomyces cerevisiae*

**DOI:** 10.1371/journal.pgen.1005420

**Published:** 2015-08-04

**Authors:** Christine E. Cucinotta, Alexandria N. Young, Kristin M. Klucevsek, Karen M. Arndt

**Affiliations:** Department of Biological Sciences, University of Pittsburgh, Pittsburgh, Pennsylvania, United States of America; Harvard Medical School, UNITED STATES

## Abstract

Eukaryotes regulate gene expression and other nuclear processes through the posttranslational modification of histones. In *S*. *cerevisiae*, the mono-ubiquitylation of histone H2B on lysine 123 (H2B K123ub) affects nucleosome stability, broadly influences gene expression and other DNA-templated processes, and is a prerequisite for additional conserved histone modifications that are associated with active transcription, namely the methylation of lysine residues in H3. While the enzymes that promote these chromatin marks are known, regions of the nucleosome required for the recruitment of these enzymes are undefined. To identify histone residues required for H2B K123ub, we exploited a functional interaction between the ubiquitin-protein ligase, Rkr1/Ltn1, and H2B K123ub in *S*. *cerevisiae*. Specifically, we performed a synthetic lethal screen with cells lacking *RKR1* and a comprehensive library of H2A and H2B residue substitutions, and identified H2A residues that are required for H2B K123ub. Many of these residues map to the nucleosome acidic patch. The substitutions in the acidic patch confer varying histone modification defects downstream of H2B K123ub, indicating that this region contributes differentially to multiple histone modifications. Interestingly, substitutions in the acidic patch result in decreased recruitment of H2B K123ub machinery to active genes and defects in transcription elongation and termination. Together, our findings reveal a role for the nucleosome acidic patch in recruitment of histone modification machinery and maintenance of transcriptional integrity.

## Author Summary

Chromatin, a complex of DNA wrapped around histone proteins, impacts all DNA-templated processes, including gene expression. Cells employ various strategies to alter chromatin structure and control access to the genetic material. Nucleosomes, the building blocks of chromatin, are subject to a myriad of modifications on their constituent histone proteins. One highly conserved modification with important connections to human health is the addition of ubiquitin to histone H2B. H2B ubiquitylation modulates chromatin structure during gene transcription and acts as a master regulator for downstream histone modifications. The proteins that promote H2B ubiquitylation have been identified; however, little is known about how these proteins interface with the nucleosome. Here, we exploited the genetic tools of budding yeast to reveal features of the nucleosome that are required for H2B ubiquitylation. Our genetic screen identified amino acids on the nucleosome acidic patch, a negatively charged region on the nucleosome surface, as being important for this process. The acidic patch is critical for regulating chromatin transactions, and, in our study, we identified roles for the acidic patch throughout transcription. Our data reveal that the acidic patch recruits histone modifiers, regulates histone modifications within the H2B ubiquitylation cascade, and maintains transcriptional fidelity.

## Introduction

In eukaryotes, transcription and other nuclear processes take place in the context of chromatin. The basic unit of chromatin is the nucleosome, which consists of approximately 147 base pairs of DNA wrapped around a histone octamer, containing two copies of each of the four core histone proteins: H2A, H2B, H3, and H4 [1]. Histones are decorated with posttranslational modifications, which can alter chromatin architecture and recruit a wide range of proteins to the genome, thus regulating all chromatin transactions [2]. In addition to their intrinsic effects on modulating the chromatin template, certain histone modifications can promote other histone modifications, either on the same histone (*cis*-regulation) or on a different histone (*trans*-regulation) in a process termed “histone crosstalk” [3].

The monoubiquitylation of H2B on lysine 123 (H2B K123ub) in *S*. *cerevisiae* is associated with active gene transcription, impacts global nucleosome occupancy and plays important roles in transcription elongation, telomeric silencing, DNA replication, and DNA repair [4]. In yeast, this modification is catalyzed by the ubiquitin-protein ligase Bre1 and the ubiquitin-conjugating enzyme Rad6 [5–7]. In humans, the analogous lysine, H2B K120, is ubiquitylated by RNF20/RNF40 and RAD6A/RAD6B [8,9]. In one of the best-studied examples of histone crosstalk, H2B K123ub is required for other histone modifications associated with active transcription: H3 K4 and H3 K79 di- and tri-methylation [10–12]. H3 K4 dimethylation, which is enriched at the 5'-ends of coding regions, and H3 K4 trimethylation, which is associated with active promoters, regulate histone acetylation patterns on genes by directing the recruitment of histone acetyltransferases and histone deacetylases [13]. H3 K79 methylation occurs across active genes, and dimethylation of this residue locally alters the nucleosome surface [14,15]. All of these histone modifications are conserved in higher eukaryotes, and disruption of these modifications can result in a range of human diseases, including cancer [16].

In addition to Rad6 and Bre1, several protein complexes that regulate transcription elongation and nucleosome dynamics are required for wild-type levels of H2B K123ub. These include the Bur1-Bur2 cyclin-dependent kinase complex and the FACT histone chaperone complex [17–19]. Additionally, the Polymerase Associated Factor 1 complex (Paf1C), which travels with RNA pol II and Spt5 during transcription elongation, promotes H2B K123ub through the Rtf1 subunit of the complex [20–23]. While protein complexes that promote H2B K123ub have been identified, little is known about how the nucleosome itself promotes H2B K123ub.

We previously reported that the ubiquitin-protein ligase Rkr1/Ltn1 is required for the viability of yeast cells that lack the *RTF1* gene or harbor an amino acid substitution for H2B K123 that prevents ubiquitylation (H2B-K123R) [24]. Rkr1/Ltn1 associates with ribosomes and degrades nonstop proteins [25,26]. The genetic interactions between *rtf1∆*, *H2B-K123R*, and *rkr1∆* suggest a requirement for the quality control functions of Rkr1 in the absence of an intact H2B ubiquitylation pathway. We reasoned that the negative genetic interactions between *rkr1∆* and H2B-K123R could be exploited to identify histone residues that are required for H2B K123ub. Using a genetic screen, we identified H2A and H2B residues required for proper H2B K123ub and downstream histone modifications. Many of these residues map to the acidic patch on the surface of H2A. We found that amino acid substitutions in the acidic patch cause defects in the recruitment of the H2B K123ub machinery to active genes, an accumulation of read-through transcripts, and altered transcription elongation efficiency *in vivo*. Interestingly, the substitutions differentially impact histone modifications downstream of H2B K123ub. Therefore, while the H2A acidic patch residues functionally converge in regulating H2B K123ub, they diverge in regulating downstream histone modifications. Our data reveal a requirement for the nucleosome acidic patch in H2B K123ub and argue that this exposed nucleosome surface serves as an important protein docking site in which individual residues uniquely contribute to the regulation of histone modifications and gene expression.

## Results

### A genetic screen to identify histone residues important for H2B K123ub

**Fig 1:**
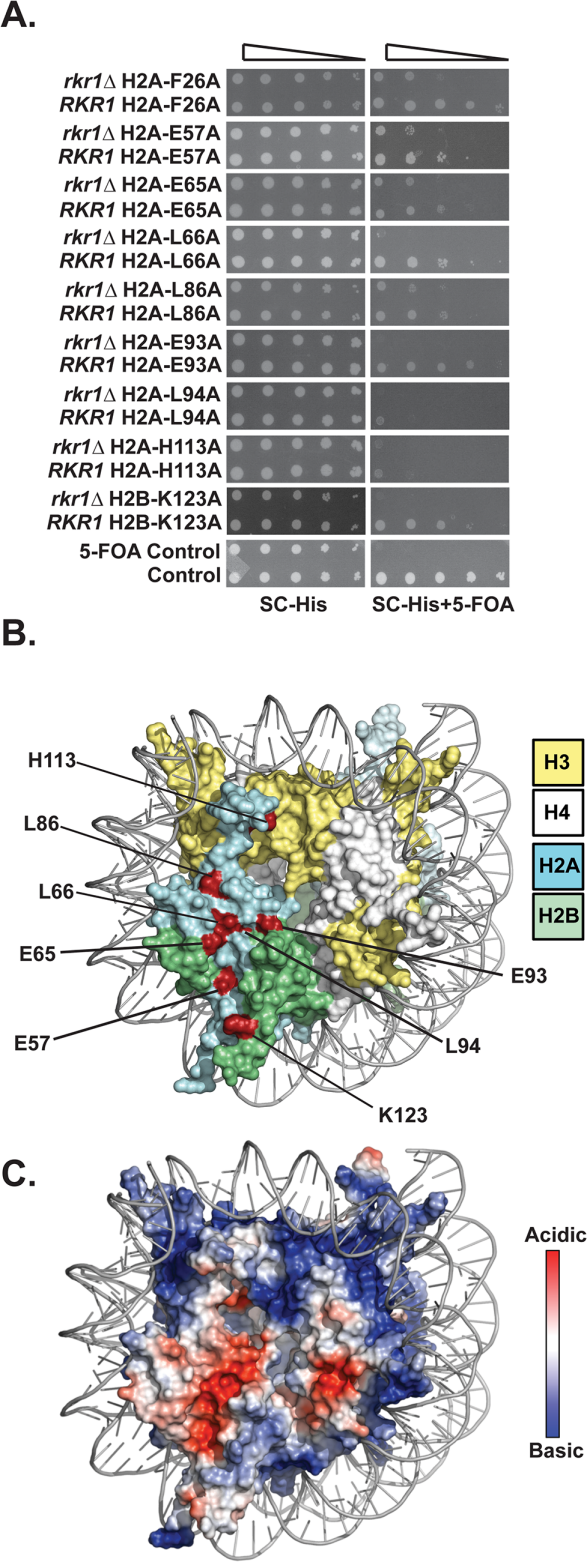
Identification of H2A and H2B residues required for growth in the absence of *RKR1*. **(A)** Synthetic lethal/sick phenotypes of *rkr1∆ hta1* and *rkr1∆ htb1* mutants were assessed through ten-fold serial dilution assays. Double mutant cells, as well as control *RKR1 hta1* and *RKR1 htb1* cells, were plated on SC-His medium as a growth control and on SC-His + 5-FOA medium to select for histone mutant plasmids and against the *URA3*-marked *HTA1-HTB1* plasmid. Library plasmids were transformed into the *rkr1∆* strain KY981 and wild-type strain KY943. KY2265 and KY2249 were used as respective negative and positive growth controls on 5-FOA plates. (**B)** X-ray crystal structure of the nucleosome, denoting histones H2A, H2B, H3, and H4 in cyan, green, yellow, and white, respectively. As depicted in red, the majority of histone residues identified in the *rkr1∆* synthetic lethality screen form a surface-exposed patch on the nucleosome. **(C)** Electrostatic potential (red is negative, blue is positive) of the nucleosome core particle. This figure was created using Pymol (PDB 1ID3 [35]).

To identify histone residues required for H2B K123ub in *S*. *cerevisiae*, we screened a comprehensive histone mutant library [27] for alanine substitutions in H2A and H2B that cause synthetic lethality or sickness when combined with a deletion of the *RKR1* gene. We previously showed that *rkr1∆* is synthetically lethal in strains carrying H2B-K123R as the only form of H2B [24]. Using a plasmid shuffle strategy, *HIS3*-marked *hta1-HTB1* or *HTA1-htb1* plasmids from the library were transformed into a *rkr1∆* deletion strain, replacing a *URA3*-marked plasmid carrying wild-type copies of *HTA1* and *HTB1*. The *URA3*-marked wild-type plasmid was counter-selected on medium containing 5-fluoroorotic acid (5-FOA). Relative to their effects on a strain containing a wild-type *RKR1* gene, nine histone mutant plasmids caused enhanced growth defects in the *rkr1∆* background (Fig 1A). Eight of the amino acid substitutions were located in H2A, and one was H2B-K123A (Fig 1A). Identification of H2B-K123A served as a validation of our screen.

Many of the residues identified in our screen cluster within the nucleosome acidic patch (Fig 1B and 1C). The acidic patch serves as a binding site for several proteins, including the H4 tail of neighboring nucleosomes [1,28–30]. In addition to those in the acidic patch, two residues, L86 and H113, reside near the docking domain of H2A [1].

**Fig 2:**
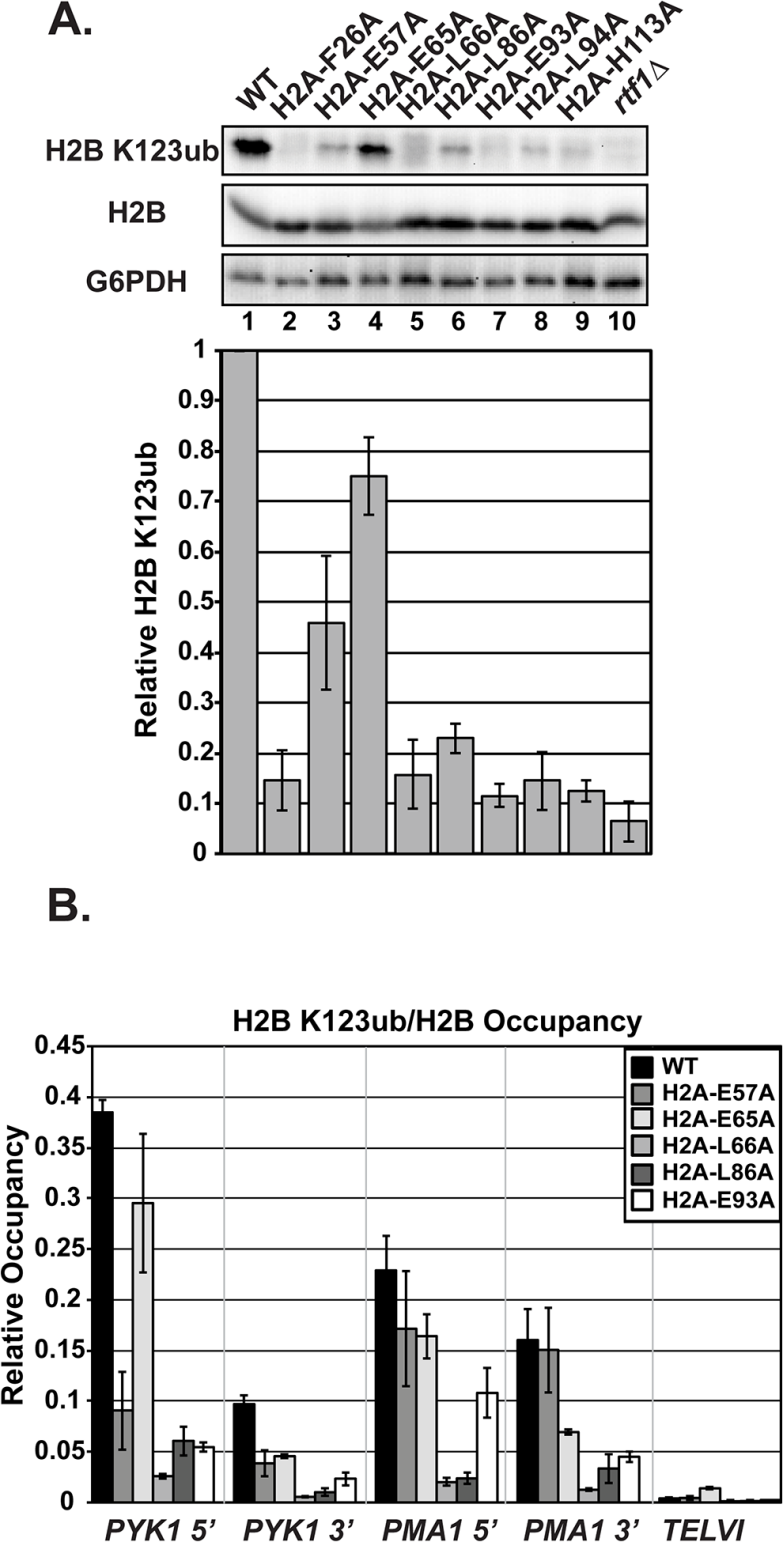
Substitutions in H2A cause H2B K123ub defects. **(A)** Western analysis of H2B K123ub, as well as total H2B and G6PDH, both of which served as loading controls. KY1599 (*rtf1∆*) was used as a negative control. The bar graph shows H2B K123ub levels normalized to total H2B levels. These relative H2B K123ub levels were normalized to the wild-type value. Error bars represent SEM of three independent experiments. **(B)** ChIP analysis of H2B K123ub occupancy at the 5'- and 3'- ends of *PYK1* and *PMA1* and at a nontranscribed region, *TELVI*. H2B K123ub ChIP values were normalized to total H2B ChIP values. The error bars represent SEM of three independent experiments.

To test if the amino acid substitutions in H2A cause H2B K123ub defects, we assessed global H2B K123ub levels by western blot analysis. Because the plasmids in the H2A and H2B mutant libraries encode FLAG-tagged H2B [27], we initially used anti-FLAG western blots to distinguish H2B K123ub from unmodified H2B as a super-shifted band. We subsequently turned to a commercial antibody against human H2B K120ub, which can specifically detect yeast H2B K123ub [31] (Fig 2). Surprisingly, this antibody did not recognize FLAG-tagged H2B K123ub to the same degree as untagged H2B K123ub in our strains, raising concerns that the FLAG tag could influence H2B ubiquitylation or our ability to detect this modification (S1 Fig). Therefore, we removed the FLAG tag from all of the plasmids carrying *hta1-HTB1* mutations identified in our screen, and we continued with these constructs for all experiments in this study. The western analysis revealed that all of the H2A mutants have reduced global H2B K123ub levels compared to the wild-type control strain; however, the different substitutions affect H2B K123ub levels to varying degrees (Fig 2A). For example, there is a striking difference in H2B K123ub levels in strains harboring substitutions of the neighboring residues H2A-E65 and H2A-L66 (Fig 2A, lanes 4 and 5). Our result reflects the H2B K123ub defect previously observed for an H2A-L66A mutant [27]; however, with removal of the FLAG tag, we now detect a defect in H2B K123ub in the H2A-E65A mutant as well.

To measure chromatin-associated levels of H2B K123ub, we performed chromatin immunoprecipitation (ChIP) analysis of H2B K123ub and total H2B at active genes (*PYK1* and *PMA1*) and, as a control, at a non-transcribed region (*TELVI*). We normalized levels of H2B K123ub to levels of total H2B to correct for any defects in H2B occupancy (Fig 2B). For these ChIP analyses, and most other experiments in this study, we focused our efforts on H2A residues E57, E65, L66, L86, and E93, because residues F26 and L94 are buried within the protein core of the nucleosome and could be impacting H2B K123ub levels indirectly (Fig 1A). We also chose not to focus on H113, because it is not conserved in higher eukaryotes. In agreement with the western analyses, the ChIP assays revealed reduced H2B K123ub levels on active genes in the H2A mutant strains (Fig 2B). However, gene-specific defects are evident. For example, the E57A substitution causes an H2B K123ub defect at *PYK1* but not *ADH1* or *PMA1* (Figs 2B and S2). Together these data demonstrate that the nucleosome acidic patch promotes H2B K123ub globally and at specific genes.

### The H2A mutants have reduced histone occupancy but do not show dramatic transcriptional changes at loci that are sensitive to chromatin integrity

**Fig 3:**
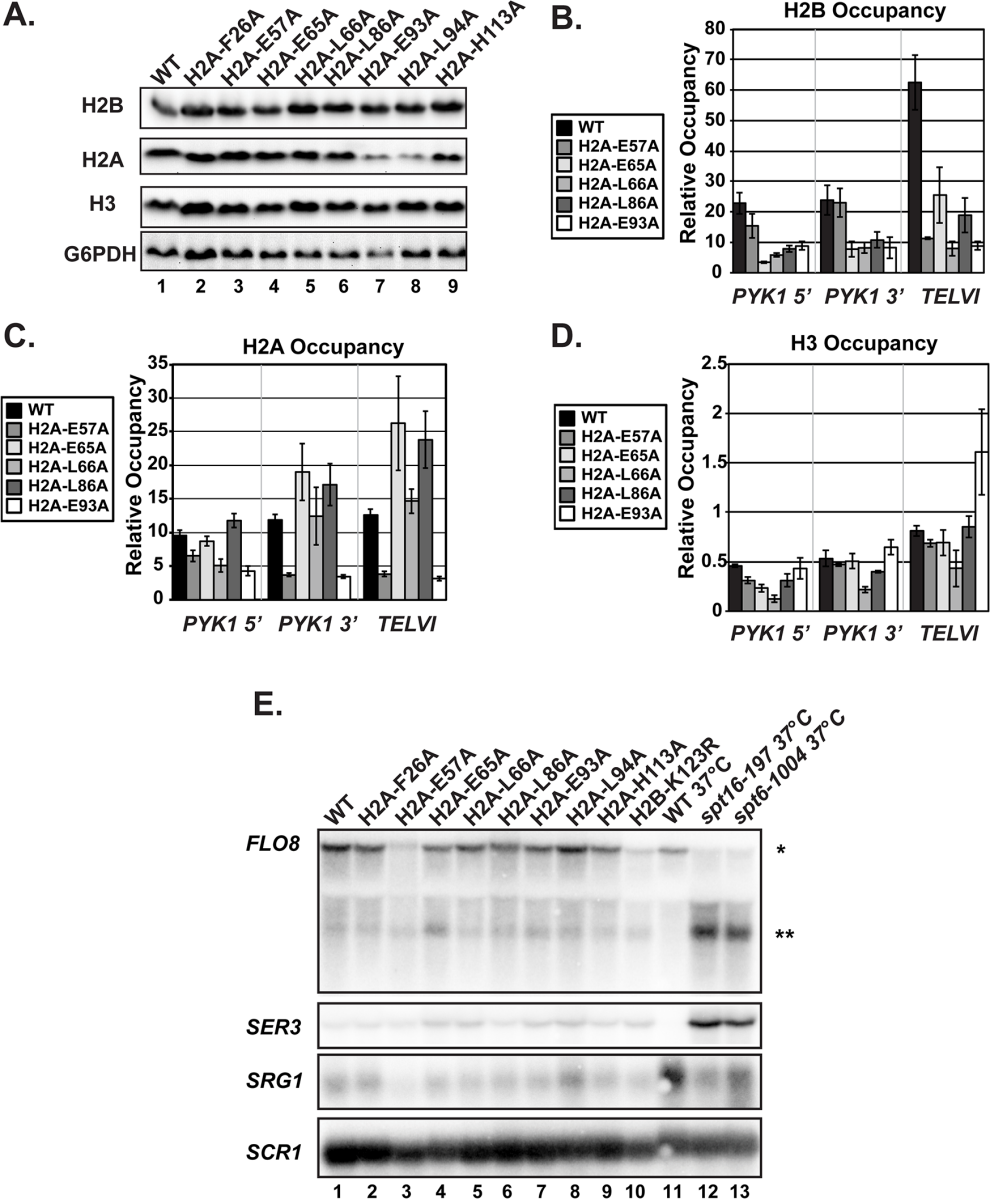
The H2A substitutions affect histone levels on genes but do not greatly affect transcription of genes that are sensitive to nucleosome occupancy. **(A)** Western analysis of H2B, H2A, and H3 levels in the H2A mutant strains. G6PDH levels served as a loading control. **(B, C, D)** Analysis of H2B (KY2674), H2A (KY2675), and H3 (KY943) occupancy at the 5’- and 3’ ends of *PYK1* and at *TELVI* by ChIP. The error bars denote SEM of three independent experiments. **(E)** Northern analysis assessing the effects of the H2A substitutions and H2B-K123R (KY2044) on *SER3*, *SRG1*, *FLO8* and *FLO8* cryptic transcript levels. Upper band (*) corresponds to the full-length *FLO8* transcript and the lower band (**) corresponds to the cryptic internally initiated transcript. The *spt6-1004* (KY2678) and *spt16-197* (KY2679) temperature-sensitive alleles serve as positive controls for cryptic initiation and *SER3* derepression and are isogenic to the wild-type strain KY2677. *SCR1* was used as a loading control.

Previous studies have shown that H2B K123ub is required for proper histone occupancy [32,33], that the docking domain of H2A is important for the association of H2A and H2B with H3 and H4 [33–35], and that the acidic patch lies at the interface of H2A and H2B [1,35]. Therefore, we examined global and local levels of histones by western analysis and ChIP, respectively (Fig 3). Global levels of H2B, H3, and H2A were unaffected in the mutants, with two exceptions (Fig 3A). The two exceptions, H2A-E93A and H2A-L94A, were detected at levels that were lower than wild-type H2A, indicating a potential defect in the expression, stability, or antibody recognition of these H2A mutant proteins. H2B, H2A, and H3 occupancy levels were assessed at both the highly transcribed gene *PYK1* and a non-transcribed telomeric region using ChIP analysis (Fig 3B–3D). Four of the alanine substitutions in H2A resulted in lower occupancy levels of H2B at *PYK1* (Fig 3B). H2A occupancy was not as drastically affected in the mutant strains; however, the signals for H2A-E57A and H2A-E93A enrichment were reduced at all loci tested (Fig 3C). For H2A-E93A, this could be due to reduced H2A protein levels or reduced immunoreactivity (Fig 3A). H3 occupancy levels at *PYK1* were also slightly affected in some of the mutant strains, particularly at the 5’ end of the gene (Fig 3D). Importantly, the reduced histone occupancy levels do not account for the reduced H2B K123ub levels in the H2A mutant strains, as we have normalized the H2B K123ub levels to total histone levels in our assays (Fig 2).

As an alternative measure of chromatin integrity in the histone mutant strains, we used northern analysis to monitor transcription of the *SER3* and *FLO8* genes, which can serve as sensitive reporters of defects in chromatin structure [36–38]. In rich media, *SER3* expression is repressed by transcription-coupled nucleosome assembly over its promoter via transcription of a noncoding RNA, *SRG1* [39,40]. Mutations in the genes encoding the histone chaperones Spt6 and Spt16 lead to strong derepression of *SER3* without decreasing *SRG1* transcription [39]. Relative to the temperature-sensitive alleles *spt6-1004* and *spt16-197*, the H2A substitutions identified in our screen do not cause strong derepression of *SER3*, suggesting that transcription-coupled nucleosome occupancy is largely intact over *SRG1* (Fig 3E).

Cryptic initiation can occur when cryptic promoters within coding regions are unveiled by perturbations in nucleosome occupancy or histone modifications [36–38]. To assess cryptic initiation in the H2A mutants, we performed northern analysis of the *FLO8* gene, using *spt6-1004* and *spt16-197* as positive controls for cryptic initiation (Fig 3E). Relative to these control strains, the H2A mutants generate only very low levels of cryptic transcripts at *FLO8* (Fig 3E). Together, these data suggest that, although histone occupancy defects can be detected, chromatin structure is not grossly impaired in the H2A mutants.

### H2A mutants have a range of defects in histone modifications dependent on H2B K123ub

**Fig 4:**
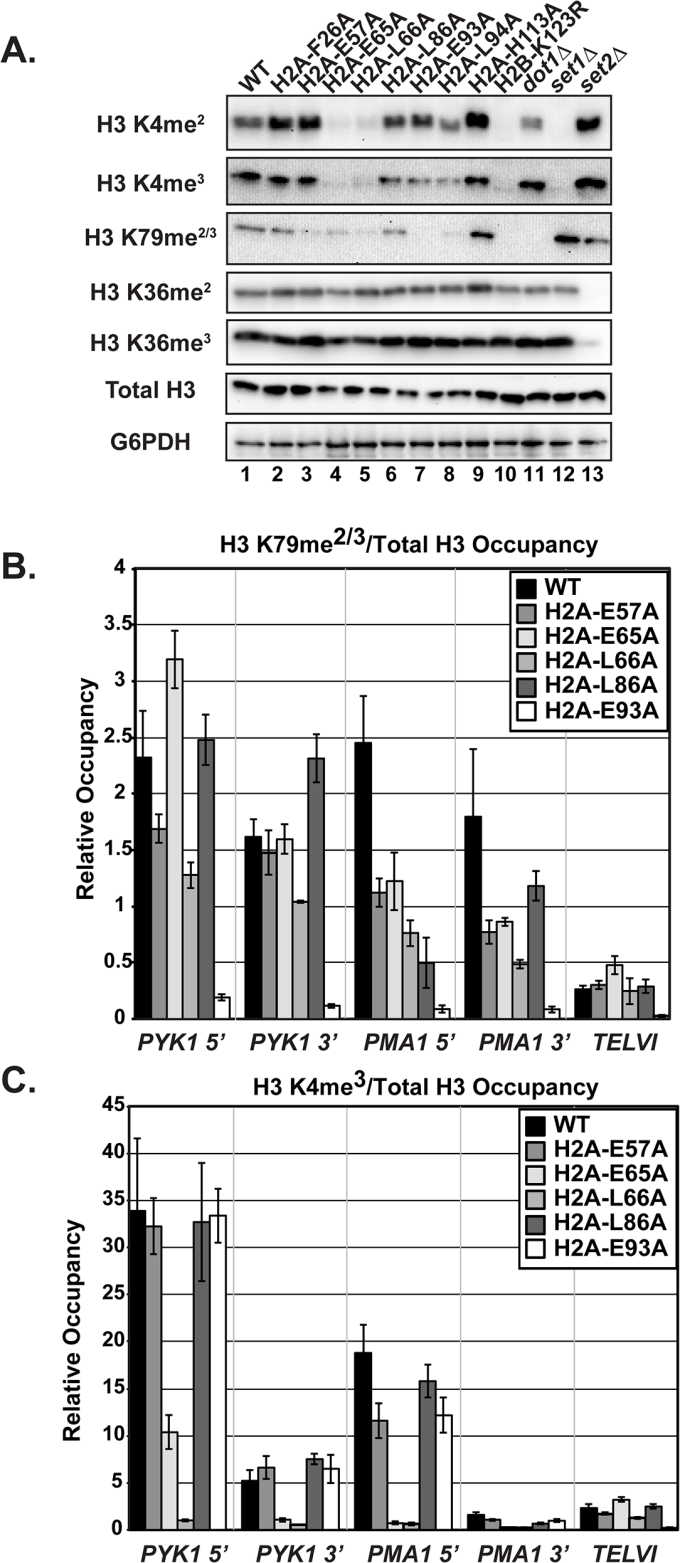
The H2A substitutions differentially affect H3 methylation. **(A)** Western blots were probed with antibodies to detect di- and tri-methylation of H3 K4, K36, and K79 as indicated. Total H3 and G6PDH levels were used as loading controls. Strains lacking *SET1* (KY1715), *DOT1* (KY1717), and *SET2* (KY1716) show the specificity of the antibodies used. **(B, C)** ChIP analysis of methylated H3 K79 and K4 at *PYK1*, *PMA1* and *TELVI*. The H3 K79 antibody used in these experiments can detect both the di- and tri-methylated states (Abcam). The error bars represent SEM of three independent experiments.

H2B K123ub is required for downstream histone modifications, including H3 K4 di- and tri-methylation (H3 K4me^2/3^), catalyzed by Set1, and H3 K79 di- and tri-methylation (H3 K79me^2/3^), catalyzed by Dot1 [10,11,13]. We therefore asked whether the H2A substitutions also cause defects in modifications downstream of H2B K123ub, using western analysis. Surprisingly, although all of the H2A mutants identified in our screen have reduced H2B K123ub levels, we observed a range of defects in H3 methylation (Fig 4). For example, two substitutions, F26A and H113A, cause no apparent defects in global H3 K4 or K79 methylation, despite dramatically reducing H2B K123ub levels (Figs 2A and 4A). In contrast, the E65A and L66A substitutions greatly reduce H3 K4 methylation and partially reduce H3 K79 methylation even though their effects on H2B K123ub levels are quite different (Figs 2A and 4A). Substitution of residues E93 and L94 to alanine resulted in a strong H3 K79 methylation defect and only slight defects in H3 K4 methylation (Fig 4A). Thus, E93 and L94 appear to selectively impact H3 K79 methylation. To determine the levels of H3 methylation on chromatin, we performed ChIP analysis of H3 K79me^2/3^ and H3 K4me^3^ at *PYK1*, *PMA1* and *TELVI* in the H2A mutant cells and normalized the data to total H3 occupancy levels (Fig 4B). The modification defects observed by ChIP mirror the global H3 methylation defects visualized by western analysis with slight differences being likely due to differences in histone occupancy levels, which were taken into account in the ChIP assay. Our results indicate that the H2A residues play unique roles in regulating histone modifications dependent on H2B K123ub.

To test whether the H2A substitutions confer other histone modification defects potentially through a general change in nucleosome structure, we performed western analysis of Set2-catalyzed H3 K36me^2^ and K36me^3^, modifications that are not strongly dependent on H2B K123ub [12,20]. None of the H2A mutants exhibited defects in H3 K36 methylation (Fig 4A). This is in agreement with previous work, which identified a distinct nucleosome surface required for H3 K36 methylation [41], and the idea that the H2A substitutions identified in our screen are largely specific to the H2B K123ub cascade.

Previous studies have shown that H3 K4me^3^ and H2B K123ub are required for proper transcription termination of small nucleolar RNAs (snoRNAs) through the Nrd1-Nab3-Sen1 pathway [42–44]. However, little is known about how these histone modifications or other nucleosome residues affect transcription termination. To assess transcription termination in our mutants, we performed RT-qPCR analysis on four snoRNA genes that are affected by histone modifications [44]. For these assays, we used probes that hybridize to the intergenic region between the snoRNA gene and the downstream gene. Detection of a PCR product is a measure of transcription in the region downstream of the snoRNA terminator (S3 Fig). The RT-qPCR analysis indicates that the H2A acidic patch residues are required for proper transcription termination at the four snoRNA loci (S3 Fig). Previous work described snoRNA termination to be differentially sensitive to disruption of H2B K123ub: *SNR47* required H2BK123ub for proper termination whereas *SNR48* was relatively insensitive to the absence of this mark [44]. The mutants identified in our screen, which all have abrogated H2B K123ub, have termination defects at both loci, indicating that the mechanistic basis for read-through of these terminators could be downstream of H2B K123ub (S3 Fig).

### Deletion of *UBP8* increases H2B K123ub and H3 methylation in a subset of the mutants

**Fig 5:**
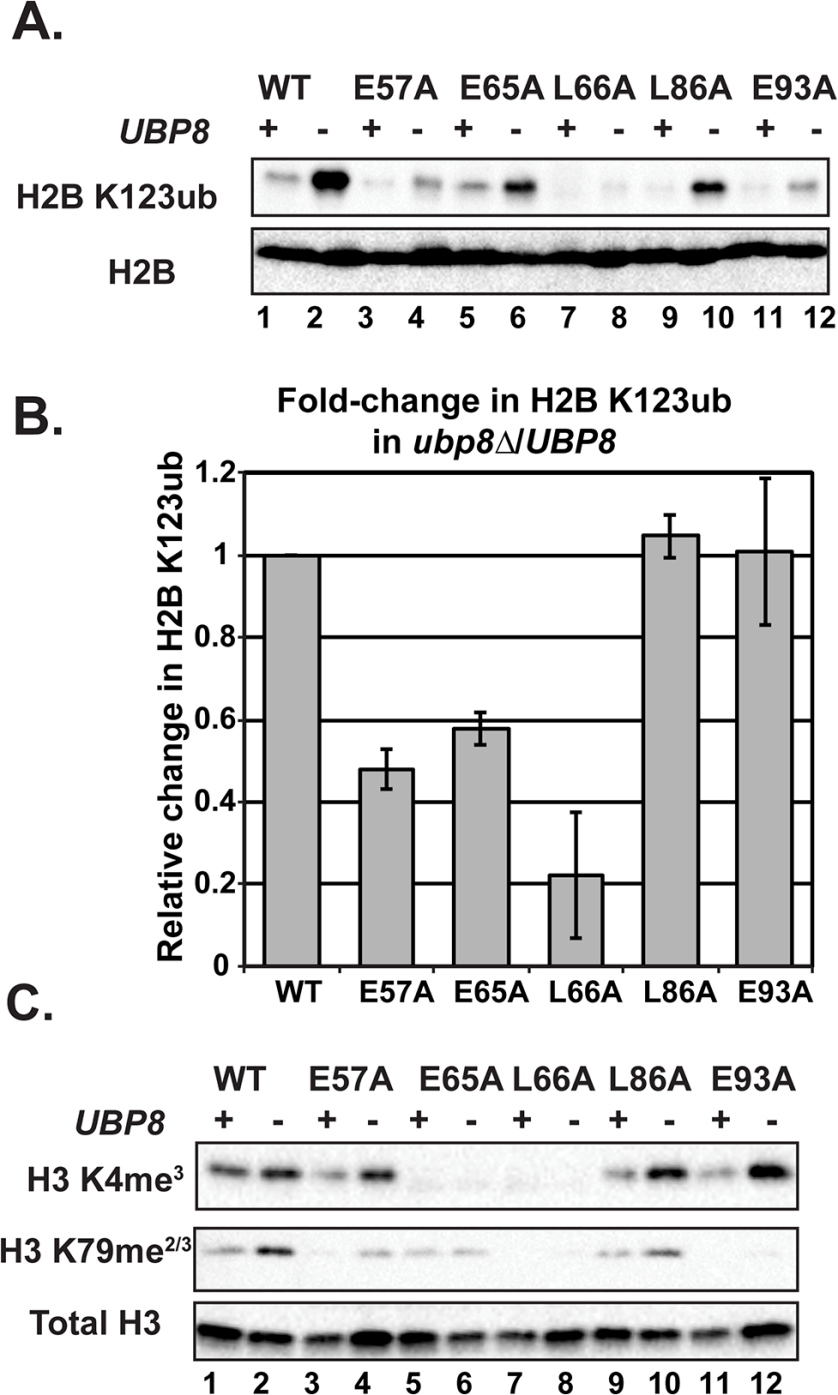
Deletion of *UBP8* variably affects the recovery of H2B K123ub, H3 K4me^3^, and H3 K79me^2/3^ in the H2A mutants. **(A)** Western analysis of H2B K123ub and total H2B in the indicated wild-type and H2A mutant strains. **(B)** The relative levels of H2B K123ub between *ubp8∆* (KY2086) and *UBP8* (KY943) backgrounds are shown. The ratio of H2B K123ub in *ubp8∆* to H2B K123ub in *UBP8* was calculated after normalizing to total H2B levels. To determine the fold change of H2B K123ub levels between H2A mutants, these ratios were normalized to the wild-type H2A background. Error bars represent SEMs of three biological replicates. (C) Western analysis of H3 K4me^3^ and H3 K79me^2/3^ levels in H2A mutants in the presence or absence of *UBP8*. Total H3 served as a loading control.

H2B K123ub is a transient histone modification; therefore one possible explanation for reduced H2B K123ub levels in the H2A mutants could be through decreased stability of the mark through the enhanced action of a ubiquitin-specific protease. The removal of H2B K123ub is due to the actions of two ubiquitin-specific proteases Ubp8 and Ubp10 [45–48]. To test whether the H2B K123ub deficiency observed in the H2A mutants is through decreased stability of the modification, we performed western blot analysis of H2B K123ub levels in strains that contain the H2A substitutions and are deleted for *UBP8*. Upon deletion of *UBP8*, the fold recovery of H2B K123ub levels was comparable to that of wild-type cells for the H2A-L86A and H2A-E93A mutants, suggesting that the H2B K123ub defect in these mutants is at least partially due to decreased stability of the mark (Fig 5A and 5B). For the H2A-E57A, H2A-E65A, and H2A-L66A mutants, deletion of *UBP8* did not fully rescue H2B K123ub levels (Fig 5A and 5B). The most drastic effect was that of H2A-L66A, where little to no H2B K123ub was restored. Therefore, for these mutants, and especially H2A-L66A, the defect in H2B K123ub is likely due to a failure of the ubiquitylation machinery to fully establish the mark. An alternative, but not mutually exclusive explanation, is that E57, E65, and L66 could form a surface required for Ubp8 function or recruitment, as these three residues reside near each other on the nucleosome structure (Fig 1B).

To test the extent to which H2B K123ub and downstream H3 methylation events are coupled in the H2A mutant strains, we measured H3 K4me^3^ and H3 K79me^2/3^ levels in the presence and absence of *UBP8*. Upon deletion of *UBP8*, H3 K4me^3^ and H3 K79me^2/3^ increased in the wild-type strain and in the H2A-L86A mutant (Fig 5C). When normalized to total H3 levels, an increase in H3 K4me^3^ and K79me^2/3^ levels was not detected in the H2A-E57A mutant upon deletion of *UBP8*, which is consistent with the poor recovery in H2B K123ub in this strain (Fig 5). This result suggests there is a correlation between K123ub and downstream marks in the H2A-E57A mutant when *UBP8* is deleted. Similarly, for the H2A-L66A mutant, no recovery of the methyl marks was observed in the *ubp8∆* background, which corresponds to the severe defect in K123ub in this mutant. This observation is consistent with the idea that the establishment of H2B K123ub is the primary defect in this mutant. For the E65A mutant, H3 K4me^3^ levels were extremely low in both the presence and absence of Ubp8, even though H2B K123ub levels were substantially recovered in the *ubp8∆* background. This observation suggests that the E65A substitution prevents proper H3 methylation possibly by disrupting a functional interaction with the Set1/COMPASS complex. Finally, in agreement with our western and ChIP results (Fig 4), the E93A mutant appears most defective in supporting H3 K79 methylation, as deleting *UBP8* elevated H3 K4me^3^ levels to a greater extent than H3 K79me^2/3^ levels in this strain.

### H2A mutants have defects in recruitment of histone modification and transcription elongation machinery to active genes

**Fig 6:**
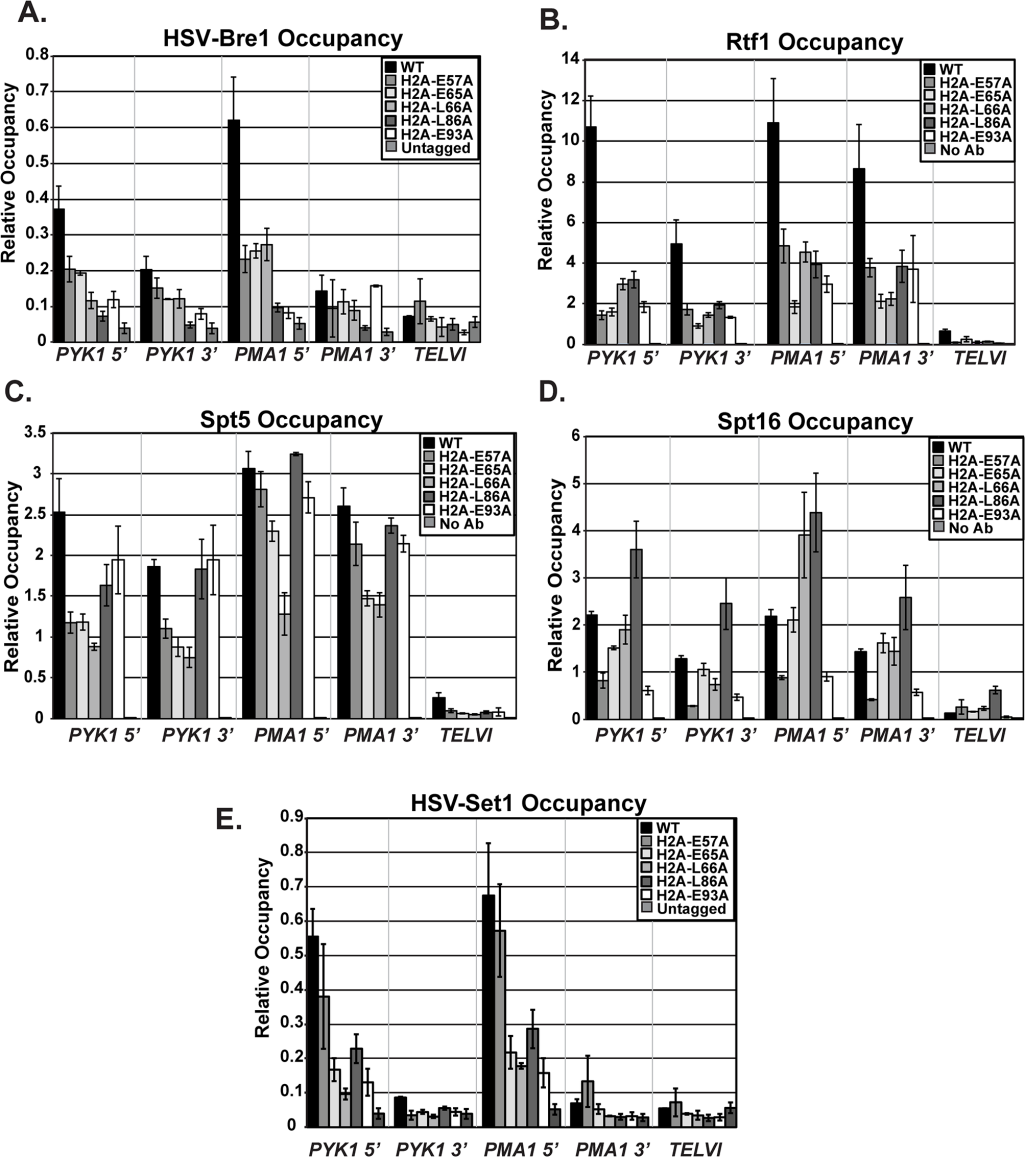
Recruitment of histone modification and elongation machinery is impaired in the H2A mutants. ChIP analyses of HSV-Bre1 (KY2674) **(A)**, Rtf1 (KY2674) **(B)**, Spt5 (KY943) **(C)**, Spt16 (KY2675) **(D)**, and HSV-Set1 (KY2719) **(E)** at the 5’- and 3’-ends of transcribed loci (*PYK1* and *PMA1*) and at *TELVI*. The error bars represent SEM of three independent experiments.

In addition to decreased stability of the ubiquitylation mark conferred by Ubp8, the reduction in histone modification levels in the H2A mutants could be due to impaired recruitment of the modification enzymes required for the H2B K123ub cascade, such as the ubiquitin-protein ligase Bre1. To analyze the effects of the H2A substitutions on recruitment of Bre1 to actively transcribed genes, we performed ChIP analysis of HSV-tagged Bre1 (Fig 6A). All five of the H2A mutants tested showed reduced recruitment of HSV-Bre1 to *PYK1* and *PMA1*, particularly at their 5’ ends (Fig 6A). With the exception of the H2A-E57A mutant, Bre1 occupancy was also reduced at *ADH1* (S2 Fig). As expected, HSV-Bre1 levels at the non-transcribed *TELVI* region were similar to those of the untagged control strain. Also in agreement with previous observations [7], Bre1 levels at the 5’ ends of *PMA1* and *PYK1* were higher than those at the 3’ ends of the genes. To determine whether reduced levels of HSV-Bre1 could account for the reduced HSV-Bre1 occupancy in the H2A mutant strains, we performed western analysis. Our results show that total HSV-Bre1 levels in the H2A mutants are similar to those in a wild-type strain (S4 Fig). These results indicate that residues in the H2A acidic patch are required for proper Bre1 recruitment to active genes.

The Paf1C subunit, Rtf1, has been implicated in the recruitment of the H2B ubiquitylation machinery during transcription [49]. We therefore used ChIP analysis to test whether the H2A residues that are important for Bre1 recruitment are also important for Rtf1 occupancy on active genes. Our ChIP results demonstrate a significant reduction in Rtf1 levels at *PYK1*, *PMA1*, and *ADH1* in the H2A mutant strains (Figs 6B and S2). To rule out the possibility that the reduced Rtf1 occupancy is a result of lower protein levels, we measured global Rtf1 levels by western analysis. This analysis showed that Rtf1 levels are unaffected in the H2A mutants, indicating that reduced Rtf1 expression is not the cause of the H2B K123ub defect (S4 Fig). Overall, the occupancy levels of HSV-Bre1 and Rtf1 correlated with H2B K123ub levels in some cases but not others. For example, the H2A-E57A mutant shows reduced HSV-Bre1 and Rtf1 occupancy but normal levels of H2B K123ub at the *PMA1* locus. It is possible that small levels of Bre1/Rad6 and Rtf1 are sufficient to promote H2B K123ub at *PMA1* in this mutant. Alternatively, decreased Ubp8 levels or activity could compensate for reduced Bre1 recruitment. We attempted to test this idea by ChIP but were unable to reliably measure Ubp8 occupancy in our strains.

We previously demonstrated that recruitment of Paf1C to coding regions is mediated through a direct physical interaction between Rtf1 and the elongation factor Spt5 [50,51]. Therefore, it is possible that the lower Rtf1 and Bre1 occupancy levels in the H2A mutant strains reflect impaired recruitment of the transcription elongation machinery. To test this idea, we performed ChIP analysis of Spt5, Spt16, and Pol II occupancy at *PYK1*, *PMA1*, and *TELVI* in the histone mutant strains (Figs 6C and 6D and S5). We observed gene and allele specific defects in Spt5 occupancy, with the E57A, E65A, and L66A substitutions causing reduced Spt5 occupancy particularly at *PYK1*. However, the levels of Spt5 occupancy largely mirrored Pol II occupancy levels, suggesting that the effects of the H2A substitutions on Spt5 recruitment are likely to be indirect. We also assessed the effects of the H2A substitutions on recruitment of the FACT complex member Spt16 (Fig 6D), which is required for proper histone occupancy and H2B K123ub [18,19,52]. Interestingly the substitution within the docking domain, H2A-L86A, of the nucleosome exhibited increased Spt16 occupancy at all tested loci. In contrast, substitutions E57A and E93A led to reduced Spt16 occupancy, suggesting that, for these H2A mutants, a defect in Spt16 recruitment may be a contributing factor to the reduced H2B K123ub levels and lower histone occupancy levels (Fig 3B and 3C). Global levels of Spt5 and Spt16 were not strongly affected, as judged by western analysis (S5 Fig).

Because the histone mutants have defects in H3 K4 methylation (Figs 4 and 5C), the acidic patch residues may be required for recruitment of the H3 K4 methyltransferase Set1. To test this, we performed ChIP analysis of HSV-tagged Set1 in the H2A mutants (Fig 6E). With the exception of E57A, all of the substitutions affect occupancy of HSV-Set1. However, after normalizing the H3 K4me^3^ occupancy levels to H3 occupancy levels, only the E65A and L66A substitutions cause a strong defect in H3 K4me^3^ (Fig 4C). We thus conclude that HSV-Set1 recruitment may be impacted by the occupancy levels of H2B K123ub and H3. For the E65A mutant, the severity of the H3 methylation defect and lack of restoration of H3 K4me^3^ upon deletion of *UBP8* suggests that E65 may play a more direct role in promoting H3 K4 methylation. We did not observe a reduction in HSV-Set1 levels in the H2A mutants (S6 Fig), as has been reported to occur when H3 K4 cannot be methylated [53,54]. It is possible that the H2A mutants lack the ability to regulate Set1 levels.

### The H2A substitutions reduce the efficiency of Pol II elongation

**Fig 7:**
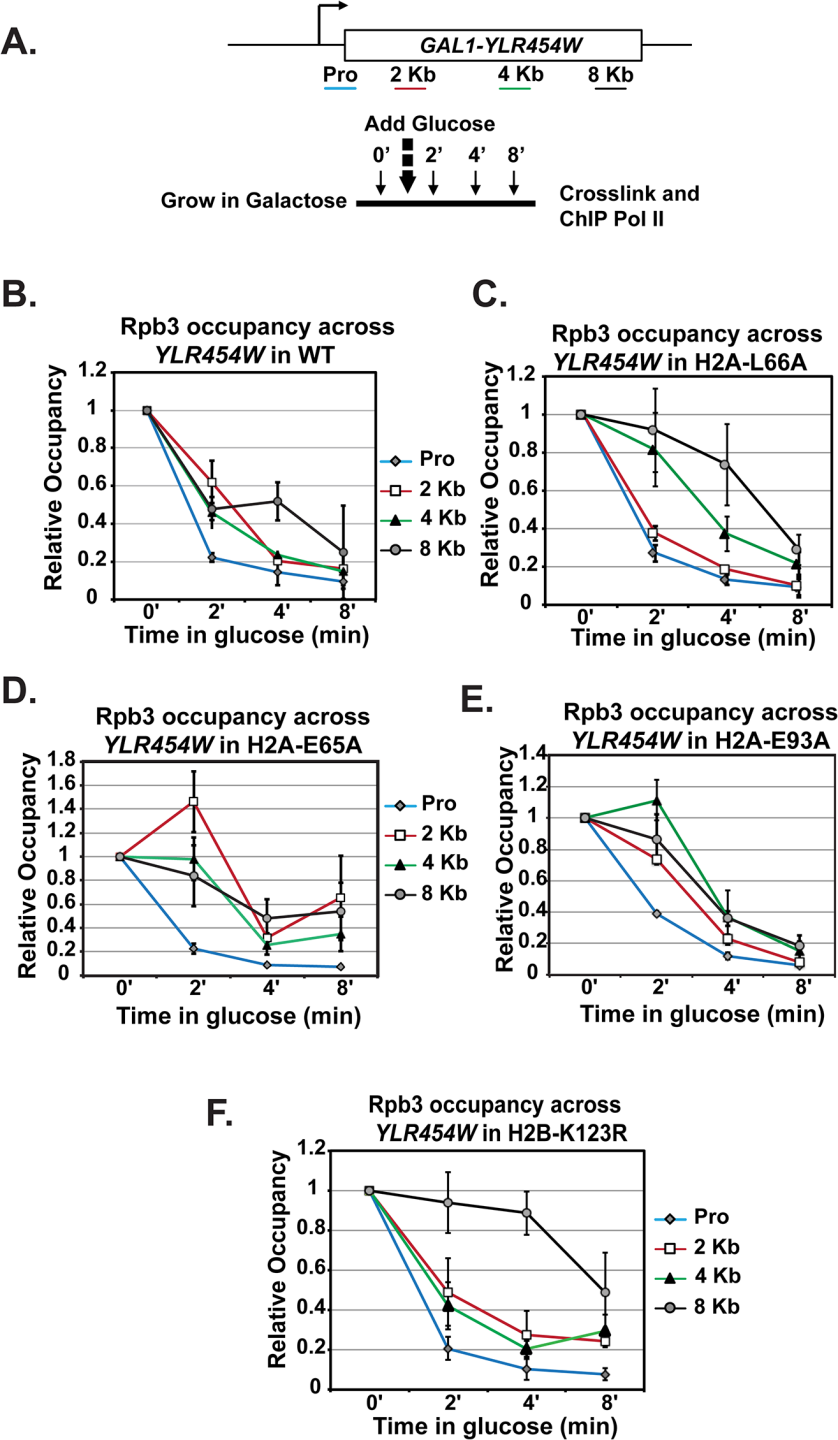
Transcription elongation is affected by substitutions in the nucleosome acidic patch. **(A)** Diagram of experimental procedure. Cells were grown in medium containing 2% galactose (zero time point) and then 2% glucose was added to shut off transcription. Samples were taken at zero, two, four, and eight-minute time points for cross-linking. ChIP of the Rpb3 subunit of Pol II across *YLR454W* was performed in **(B)** wild type (WT), **(C)** H2A-E65A, **(D)** H2A-L66A, **(E)** H2A-E93A, and **(F)** H2B-K123R strains, which were transformants of KY2676. Values were normalized to the zero time point for each locus. Error bars represent SEM of three biological replicates.

Because the H2A mutants exhibit reduced levels of transcription elongation-coupled histone modifications, we asked whether the acidic patch substitutions alter the efficiency of transcription elongation. To assess transcription elongation efficiency *in vivo* we used a well-established galactose-controlled system to shut off transcription of a gene and measure occupancy of Pol II during the last wave of transcription [55–57]. This system incorporates the *GAL1* promoter upstream of the non-essential gene *YLR454W*. Cells were grown in 2% galactose to activate the gene and 2% glucose was added to the cultures to prevent further initiation events. Samples were taken at different time points to determine "snap-shots" of Pol II density at four regions of *YLR454W* by ChIP (Fig 7A). In wild-type cells, Pol II rapidly cleared the *YLR454W* coding region, as previously described [55–57] (Fig 7B). In the H2A mutants, however, the rate and/or processivity of Pol II elongation was reduced. The most dramatic effect was observed with the H2A-L66A mutant, where Pol II density persisted at the 4 Kb and 8 Kb locations relative to the wild-type kinetics (Fig 7C). The H2A-E65A mutant also exhibited a delay in Pol II passage, with occupancy persisting at multiple locations throughout the time course (Fig 7D). The H2A-E93A mutant exhibited a slightly different and more modest elongation defect (Fig 7E). Collectively these data reveal an important role for the nucleosome acidic patch in promoting efficient transcription elongation.

Because the H2A acidic patch mutants have defects in H2B K123ub, we wanted to determine whether the *in vivo* elongation defects correlated with the loss of H2B K123ub. To begin to address this, we performed a similar analysis on H2B-K123R cells (Fig 7F). Interestingly, Pol II elongation efficiency was reduced in the H2B-K123R mutant, as indicated by persistent enrichment toward the 3’ end of the gene. These data indicate that residues within the acidic patch, at least partly through their role in promoting H2B K123ub, are important for transcription elongation efficiency *in vivo*.

## Discussion

In this study, we exploited a genetic interaction between the H2B ubiquitylation pathway and the protein quality control factor Rkr1 to identify residues in H2A and H2B that are required for H2B K123ub. We identified eight residues in H2A that, when changed to alanine, cause defects in H2B K123ub (Fig 2). Most of these residues map to the acidic patch on the nucleosome (Fig 1B), which plays critical roles in several important nuclear processes. Indeed, as shown through structural studies, the acidic patch serves as a direct binding platform on the nucleosome for a variety of proteins that affect transcription, chromatin structure, and chromosome segregation. These proteins include the Latency-Associated Nuclear Antigen (LANA) peptide from Kaposi’s sarcoma virus, the Regulator of Chromatin Condensation 1 protein (RCC1), the Bromo-Associated Homology (BAH) domain of Sir3, and the centromere binding protein CENP-C [28,30]. Additionally, as shown through functional studies and a recently published structure of the Polycomb Repressive Complex 1 ubiquitylation module in complex with a nucleosome, the acidic patch interacts with ubiquitin-protein ligases that target H2A [58–60].

Despite the importance of H2B K123ub in regulating gene expression, nucleosome stability, and genic patterns of histone methylation and acetylation, little is known about how the enzymatic machinery for H2B K123ub interfaces with the nucleosome. In a recent study, a basic region of the RING domain of Bre1 was shown to be important for interacting with the nucleosome [61]. Here, we show that nucleosome acidic patch mutants have impaired chromatin occupancy of the ubiquitin-protein ligase Bre1 and the Paf1C subunit Rtf1. The mechanism by which Rtf1 is required for H2B K123ub is largely undefined, although a recent study indicated a role for Rtf1 in stabilizing Bre1 protein levels [31]. In our H2A mutant strains, global protein levels of Bre1 are similar to those in a wild-type strain. This observation, together with our ChIP studies on Bre1 and Rtf1, suggests that the nucleosome acidic patch plays an active role in promoting H2B K123ub. A previous study found that the N-terminus of H2A, the H2A repression (HAR) domain, is also required for H2B K123ub. However, recruitment of the H2B K123ub machinery was not affected in the H2A N-terminal tail mutant [62]. It is possible, then, that the acidic patch could recruit the H2B K123ub machinery to chromatin, potentially through a direct interaction with Bre1 and/or Rtf1, while the HAR domain stimulates enzyme activity.

In light of previous work showing that Paf1C recruitment is governed by a direct physical interaction between Rtf1 and the phosphorylated C-terminal region of the elongation factor Spt5 [50,51,63,64], we were surprised that the H2A substitutions identified in our screen caused a loss in Rtf1 occupancy without a corresponding loss in Spt5 recruitment. However, it was recently shown that the human homolog of Bre1, RNF20/40, promotes recruitment of PAF1 to chromatin in human cells [65]. In addition, binding of human Paf1 to histone-like proteins and nucleosomes has been reported [66,67]. These observations align with our results and indicate that multiple interactions can mediate or stabilize the interaction between Paf1C and chromatin. Alternatively, given the importance of Spt5 phosphorylation in mediating the interaction between Rtf1 and Spt5 [50,51,63,64], it is also possible that the H2A mutants are indirectly affecting Spt5 phosphorylation. Finally, we also note that Rtf1 recruitment defects could be due to the combined effect of the individual, and relatively modest, defects in Pol II, Spt5, and Spt16 occupancy (Figs 6 and S5).

The function of the ubiquitin-specific protease Ubp8 also appears to be affected by substitutions within the acidic patch (Fig 5). A recent study suggested that the acidic patch residue H2A-Y58 promotes H2B K123ub through regulating Ubp8, as deletion of *UBP8* rescued H2B K123ub in an H2A-Y58F mutant [68]. The H2A-Y58A is a lethal substitution in yeast and could not be isolated in our screen [27]. In our study, deletion of *UBP8* rescued H2B K123ub to some degree in most of our mutants, which suggests that these mutants have defects in both ubiquitylating H2B-K123 and in stabilizing the mark (Fig 5A). For the H2A-L66A mutant, the nearly complete absence of H2B K123ub in the presence or absence of *UBP8* suggests that little ubiquitin is placed on H2B-K123 such that removal of *UBP8* makes little to no difference in this mutant.

The H2A residues we identified are required for H2B K123ub-dependent H3 methylation (Fig 4). Interestingly, some mutants exhibited defects in only H3 K4 methylation or H3 K79 methylation, while others had defects in both, despite all having reduced H2B K123ub levels. These data suggest that individual residues within the acidic patch promote methylation through separate mechanisms. Substitution of neighboring residues, H2A-E65A and H2A-L66A, differentially impacted H2B K123ub levels, but both mutants had undetectable levels of H3 K4 methylation (Fig 4) [27]. It is possible that the methylation defects caused by the L66A substitution are largely due to a severe defect in the establishment of H2B K123ub in this mutant, similar to the effect of the H2B K123R mutant [69]. In contrast, the H3 K4 methylation defect of the H2A-E65A mutant may stem primarily from the reduced recruitment and/or activation of Set1. For the H2A-E65A mutant, we noted a lack of recovery of H3 K4me^3^ and H3 K79me^2/3^ when H2B K123ub levels were increased through the deletion of *UBP8* (Fig 5C). This observation suggests that E65 is important for coupling H2B K123ub to downstream H3 methylation events. Interestingly, substitution of other residues near H2B K123 has been shown to uncouple H3 methylation from H2B K123ub. For example, H2B R119 and T112, when mutated, increase H2B K123ub levels but decrease H3 K4me^3^ levels [70].

The severe deficiency in H3 K79me^2/3^ observed in the H2A-E93A mutant (Figs 4 and 5C) presents the intriguing possibility that this residue may interact with Dot1 to promote H3 K79 methylation. It is unlikely that the H3 K79 methylation defect detected in the H2A-E93A mutant is solely due to its defect in H2B K123ub, because when H2B K123ub levels are increased in the absence of *UBP8*, the increase in H3 K79 methylation is very slight (Fig 5C). Interestingly, the basic patch in the H4 tail is required for Dot1 methylase activity, but not for Dot1 recruitment [71]. Since the H4 tail interacts with the acidic patch of the nucleosome [1,29], one explanation for the H3 K79me^2/3^ defect could be that E93 is required for recruitment of Dot1, while the H4 tail stimulates Dot1 activity.

Further supporting growing indications that chromatin structure is important for proper transcription termination through the NNS pathway, the H2A mutants tested exhibited transcriptional readthrough at four *SNR* genes (S3 Fig). The magnitude of the transcriptional defect does not correlate strictly with the loss of any particular histone modification, suggesting that this phenotype may be sensitive to the combinatorial loss of several modifications and possibly other factors, such as histone occupancy and Spt16 recruitment (Figs 3 and 6D). Regardless of the mechanism, the increased levels of aberrant transcripts in the H2A mutants could provide a rationale for the synthetic growth defects observed in H2A mutants lacking *RKR1*. Rkr1 is a protein quality control factor that is involved in the degradation of aberrant proteins, including those that extend past stop codons [25,26]. The elevated synthesis of aberrant proteins, potentially as a consequence of improper transcription in the H2A mutants, could have lethal consequences for the cell [72]. The negative genetic interaction between *rkr1∆* and the histone mutants suggests that the consequences of disrupting the acidic patch extend beyond chromatin and transcription.

We assayed the effects of the H2A substitutions on transcription elongation through analysis of Pol II density during the last wave of transcription across *GAL1-YLR454W*. In this assay, the H2A-L66A mutant exhibited a strong defect in elongation efficiency and most closely mimicked the behavior of the H2B-K123R mutant. These data support the view that H2A-L66A phenocopies H2B-K123R for loss of H2B K123ub and its consequences. The H2A-E93A and H2A-E65A mutants also exhibited impaired elongation, although not to the same degree as the H2A-L66A and H2B-K123R mutants. Given the differential effects of the H2A substitutions on the histone modification levels in the cells, differences in Pol II elongation efficiency were not unexpected. Taken together, these data indicate that H2B K123ub and its effects on downstream histone modifications and nucleosome stability are important for efficient Pol II passage through chromatin.

**Fig 8:**
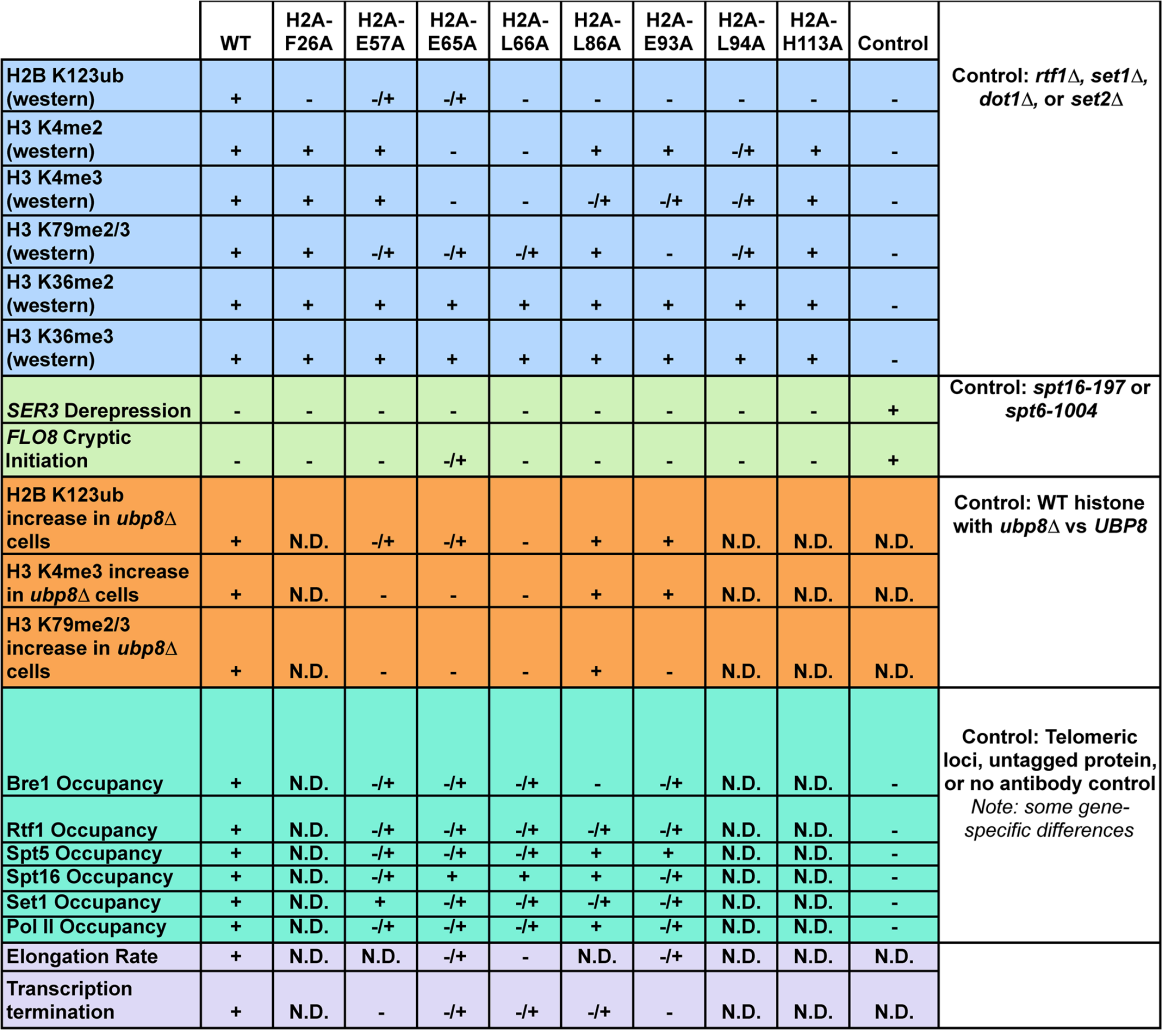
Summary of molecular defects tested in the H2A mutants. Phenotypes (listed on the left) of the histone mutants rated relative to wild type (WT) and additional controls, which are defined in the figure (right column). Molecular defects not determined for specific mutants are denoted by "N.D."

Combined, our data support a new role for the nucleosome acidic patch in transcription, specifically through the proper recruitment and/or activation of proteins that control H2B K123ub and downstream methylation events on H3. The mutations that disrupt this patch impair several transcription-related processes, including the modification of histones, recruitment of transcriptional machinery, the efficient passage of Pol II through chromatin, and transcription termination (Fig 8). Many of these transcriptional defects likely stem from the pleiotropic effects of losing the critical H2B K123ub mark. Together with recent structural studies, our results strongly suggest that the acidic patch is an interaction platform for proteins that modulate numerous chromatin transactions in eukaryotic cells. An exciting goal for future studies will be to understand how cells regulate access to this important region of the nucleosome.

## Materials and Methods

### Yeast strains and media

The *S*. *cerevisiae* strains used in this study are listed in S1 Table and are isogenic to the strain FY2, which is a *GAL2* ^+^ derivative of S288C [73]. Yeast transformations were performed as previously described [74]). With noted exceptions, experiments were performed using the strain KY943 transformed with histone mutant plasmids. To replace wild-type histone plasmids with *HIS3*-marked mutant histone plasmids, transformants were sequentially passaged three times on SC-His medium containing 2% dextrose and 0.1% 5-FOA. Unless otherwise noted, for all experiments, yeast strains were grown in SC-His medium containing 2% dextrose. HSV-Bre1 and HSV-Set1 strains contain three chromosomally located HSV tags on the N-termini of the proteins [75]. These proteins were confirmed to have proper function and expression.

### Dilution growth assays

Cells were grown to saturation at 30°C and washed with sterile water. Beginning with a cell suspension at a concentration of 1 X 10^8^ cells/mL, cells were diluted serially four times by a factor of ten in water. Two microliters of each dilution were spotted on SC-His medium and SC-His medium containing 5-FOA. Plates were incubated at 30°C for three days.

### Plasmid construction

Site-directed mutagenesis (Agilent) with primers listed in S2 Table was performed to remove the sequence encoding the FLAG tag from plasmids obtained from the H2A and H2B mutant library [27]. Plasmid sequences were confirmed by DNA sequencing. Plasmid names are given in S3 Table.

### Western blot analysis

For western analyses other than those that measure H2B K123ub, yeast cells were grown to log phase (2–3 X 10^7^ cells/mL) and lysed by bead beating in trichloroacetic acid (TCA), as described previously [76]. To make whole cell extracts for H2B K123ub analysis, cells were lysed in SUTEB buffer (10 mM Tris-HCl, pH 8.0, 1% SDS, 8 M urea, 10 mM EDTA, pH 8.0, and 0.01% bromophenol blue) [43]. Proteins were resolved on SDS-polyacrylamide gels (15% polyacrylamide for histone westerns, 10% polyacrylamide for Rtf1 and HSV-Bre1, and 8% polyacrylamide for HSV-Set1, Spt5, and Spt16 westerns) and transferred to nitrocellulose membranes. For H2B K123ub western blot analysis, proteins were transferred to PVDF membranes. Membranes were incubated with primary antibodies and then with anti-mouse or anti-rabbit secondary antibodies (GE Healthcare 1:5,000 dilution). Antibodies that recognize the following proteins or histone modifications were used: total histone H3 (1:30,000 dilution) [43], trimethylated H3 K4 (H3 K4me^3^) (Active Motif 39159, 1:2,000 dilution), H3 K4me^2^ (Millipore 07–030, 1:2000 dilution), H3 K79me^3^ (note: this antibody has been reported by the manufacturer to cross-react with H3 K79me^2^, Abcam ab2621, 1:2,000 dilution), H3 K36me^2^ (Millipore 07–369, 1:1000 dilution), H3 K36me^3^ (Abcam ab9050, 1:1000 dilution), H2A (Active Motif, 39235, 1:5,000 dilution), H2B (Active Motif, 39237, 1:5,000 dilution), HSV (Sigma-Aldrich H6030, 1:350 dilution), Spt5 (gift from Grant Hartzog, 1:1000 dilution), Spt16 (gift from Tim Formosa, 1:500 dilution), Rtf1 (1:5,000 dilution) [77], and glucose-6-phosphate dehydrogenase (G6PDH) (Sigma-Aldrich A9521, 1:30,000 dilution). An antibody against a human H2B K120ub-containing peptide (Cell Signaling 5546, 1:1000 dilution) was used to detect the analogous modification in *S*. *cerevisiae*, H2B K123ub. Proteins were visualized using enhanced chemiluminescence substrate (PerkinElmer) and either a 440 CF digital imaging station (Kodak) or a ChemiDoc XRS digital imaging station (BioRad). For western blot analysis, signals were quantified using ImageJ software and normalized to the loading control specified in the figure legend. The relative signal from the wild-type strain was set equal to one. Error bars represent standard error of the mean for three biological replicates (SEM).

### Chromatin immunoprecipitation (ChIP) and quantitative PCR assays

Chromatin immunoprecipitation (ChIP) assays were performed with 250 mL of log-phase yeast cultures (1–2 X 10^7^ cells/mL) as previously described [78]. For histone ChIPs, sheared chromatin was incubated overnight at 4°C with antibodies specific to H2B, (0.5 μl, Active Motif, 39237), human H2B K120ub (2.5 μl, Cell Signaling 5546), H3 K4me^3^ (2.5 μl, Abcam ab8580), H3 K79me^2/3^ (2.5 μl, Abcam ab2621), or total H3 (5 μl) [43]. Chromatin prepared from an H2B-K123R strain served as a specificity control for the human H2B K120ub antibody (S7 Fig). For other ChIPs, chromatin was incubated overnight at 4°C with antibodies specific to Spt16 (1 μl, gift from Tim Formosa), Spt5 (1 μl, gift from Grant Harzog), or Rpb3 (2.5 μl Neoclone W0012). Following incubation with the primary antibodies, chromatin was incubated for 2 hours at 4°C with Protein A-conjugated sepharose for all ChIPs, with the exception of Rpb3 ChIPs, for which chromatin was incubated with Protein G-conjugated sepharose (30 μl, GE Healthcare). For ChIP of HSV-Bre1 and HSV-Set1, chromatin was incubated overnight at 4°C with an antibody specific to the HSV epitope (2.5 μl, Sigma-Aldrich H6030), followed by incubation as described above. For ChIP of Rtf1, chromatin was incubated overnight at 4°C with polyclonal antisera that recognizes Rtf1 [77]. DNA was purified (Qiagen) and analyzed by qPCR using Maxima SYBR (Thermo) and primers for the 5’ coding region of *PYK1* (amplicon: +253 to +346 relative to ATG), the 3’ coding region of *PYK1* (amplicon: +1127 to +1270), the 5’ coding region of *PMA1* (amplicon: +214 to +319 relative to ATG), the 3’ coding region of *PMA1* (amplicon: +2107 to +2194), or a telomeric region of chromosome VI (chromosomal coordinates, 269495 to 269598). Occupancy levels were calculated using the primer efficiency raised to the difference between input and immunoprecipitated Ct values. Presented data are an average of two technical replicates for each of three biological replicates. The error bars indicate the standard error of the mean (SEM).

### Northern blot analysis

Total RNA was isolated from log-phase yeast cultures (1–2 X 10^7^), and 20 μg of RNA were subjected to northern blot analysis as described previously [79]. Radiolabeled DNA probes were generated through random-prime labeling reactions of PCR templates. Membranes (Gene Screen Plus, Perkin Elmer) were incubated with radiolabeled DNA probes from PCR fragments of *SCR1* (amplicon: -163 to +284 relative to the TSS), *SRG1* (amplicon: -454 to -123 relative to *SER3* ATG), *SER3* (amplicon: +111 to +1342 relative to ATG), and *FLO8* (amplicon: +1515 to + 2326 relative to ATG). See S2 Table for primer sequences. Signals were quantified using ImageJ software relative to the *SCR1* loading control, with wild type set to one. For quantification of all northern blot analyses, signals were averaged for three independent biological replicates. Error bars represent standard error of the mean (SEM).

### Quantitative real-time reverse transcription-PCR (RT-qPCR)

Total RNA was isolated as described above and then DNase treated using the Turbo DNA-free kit (Ambion, AM1907) and RNase inhibitor (Ambion, AM2682). cDNA was generated using the RETROscript kit (Ambion, AM1710) with random hexamers and oligo(dT) primers. Quantitative PCRs were performed as described above using primers specific for the regions downstream of snoRNAs (S2 Table). Signals were analyzed using the ∆∆CT method with *ACT1* used as the target gene [80]. For controls, reactions lacking reverse transcriptase or template were performed. The graphs show the results of three independent biological replicates.

## Supporting Information

S1 Fig The FLAG epitope tag impacts H2B K123ub detection. Western analysis of H2BK123ub in yeast strains carrying untagged (lane 1) or FLAG-tagged H2B (lane 2). Western blots were probed with antibodies against human H2B K120ub and total H2B, which served as a loading control.
(TIF)

S2 Fig H2B K123ub, HSV-Bre1, and Rtf1 occupancy at *ADH1*. ChIP analysis of H2B K123ub **(A)**, HSV-Bre1 **(B)**, and Rtf1 **(C)** occupancy at the *ADH1* ORF. The error bars represent SEM of three independent experiments.
(TIF)

S3 Fig H2A mutants have transcription termination defects. **(A)** Diagram of a snoRNA gene and the location of qPCR primers used to assess read-through transcription. **(B)** RT-qPCR analysis of RNA levels downstream of four different snoRNA genes in the H2A mutant strains. Transcript levels in the wild-type control strain were set to 1 and error bars represent SEM of three biological replicates.
(TIF)

S4 Fig HSV-Bre1 and Rtf1 levels are unaffected in the H2A mutants. 1-fold, 1.5-fold, and 2-fold concentrations of protein extracts were loaded on SDS polyacrylamide gels and analyzed by western blotting using anti-HSV, anti-Rtf1, and anti-G6PDH, as a loading control. Values were normalized to the initial wild-type protein concentration. Mutant strains were transformants of KY2674.
(TIF)

S5 Fig Recruitment of Pol II and levels of Spt5 and Spt16 are modestly affected in the H2A mutants. **(A)** ChIP analysis of Pol II (KY943) at the 5’- and 3’-ends of transcribed loci (*PYK1* and *PMA1*) and at *TELVI*. The error bars represent SEM of three independent experiments. Western analyses of Spt5 **(B)**, and Spt16 **(C)** to measure total protein levels in the H2A mutant cells. Values represent protein levels normalized to G6PDH with the wild-type ratio set to one.
(TIF)

S6 Fig Set1 levels are unaffected in the H2A mutants. 1-fold, 1.5-fold, and 2-fold concentrations of protein extracts were loaded on SDS polyacrylamide gels and analyzed by western blotting using anti-HSV and anti-G6PDH, as a loading control. Values were normalized to the initial wild-type protein concentration.
(TIF)

S7 Fig The antibody against human H2B K120ub can be used in yeast ChIP experiments. ChIP analysis of H2B K123ub occupancy at the 5'-end of *PYK1* and at a nontranscribed region, *TELVI*. The *TELVI* locus served as a negative control for wild-type chromatin and the K123R strain served as a negative control for the ChIP experiment.
(TIF)

S1 Table Yeast strains used in this study. (DOCX)

S2 Table Oligonucleotide primers used in this study. (DOCX)

S3 Table Plasmids used in this study. (DOCX)
