# Supplementary figures and images for: The Nucleosome Acidic Patch Regulates the H2B K123 Monoubiquitylation Cascade and Transcription Elongation in Saccharomyces cerevisiae
Source: PLoS Genet. 2015 Aug 4;11(8):e1005420. doi: 10.1371/journal.pgen.1005420 (PMC4524731; doi:10.1371/journal.pgen.1005420)

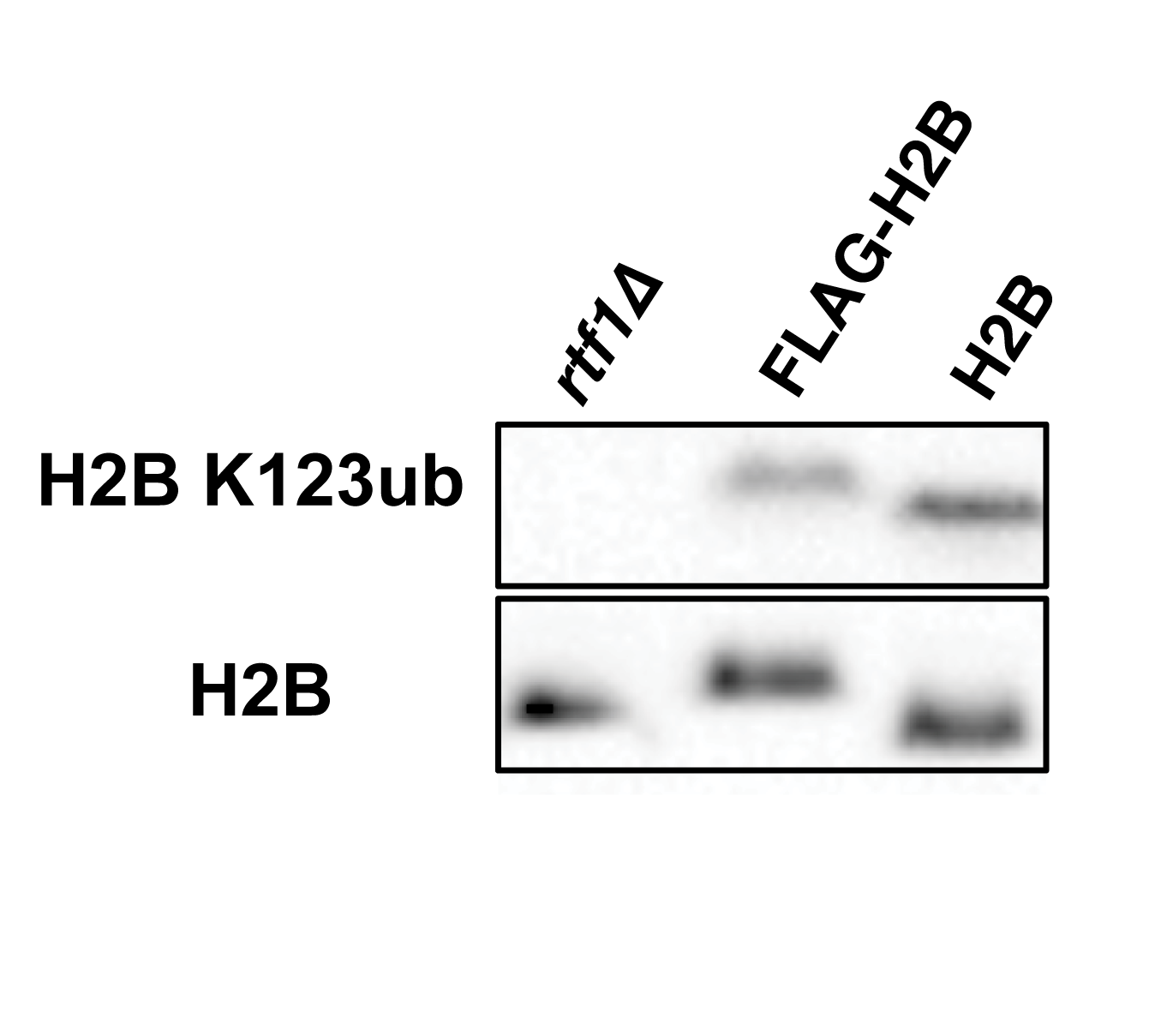

Supplement: S1 Fig — Western analysis of H2BK123ub in yeast strains carrying untagged (lane 1) or FLAG-tagged H2B (lane 2). Western blots were probed with antibodies against human H2B K120ub and total H2B, which served as a loading control. (TIF) [file pgen.1005420.s001.tif]

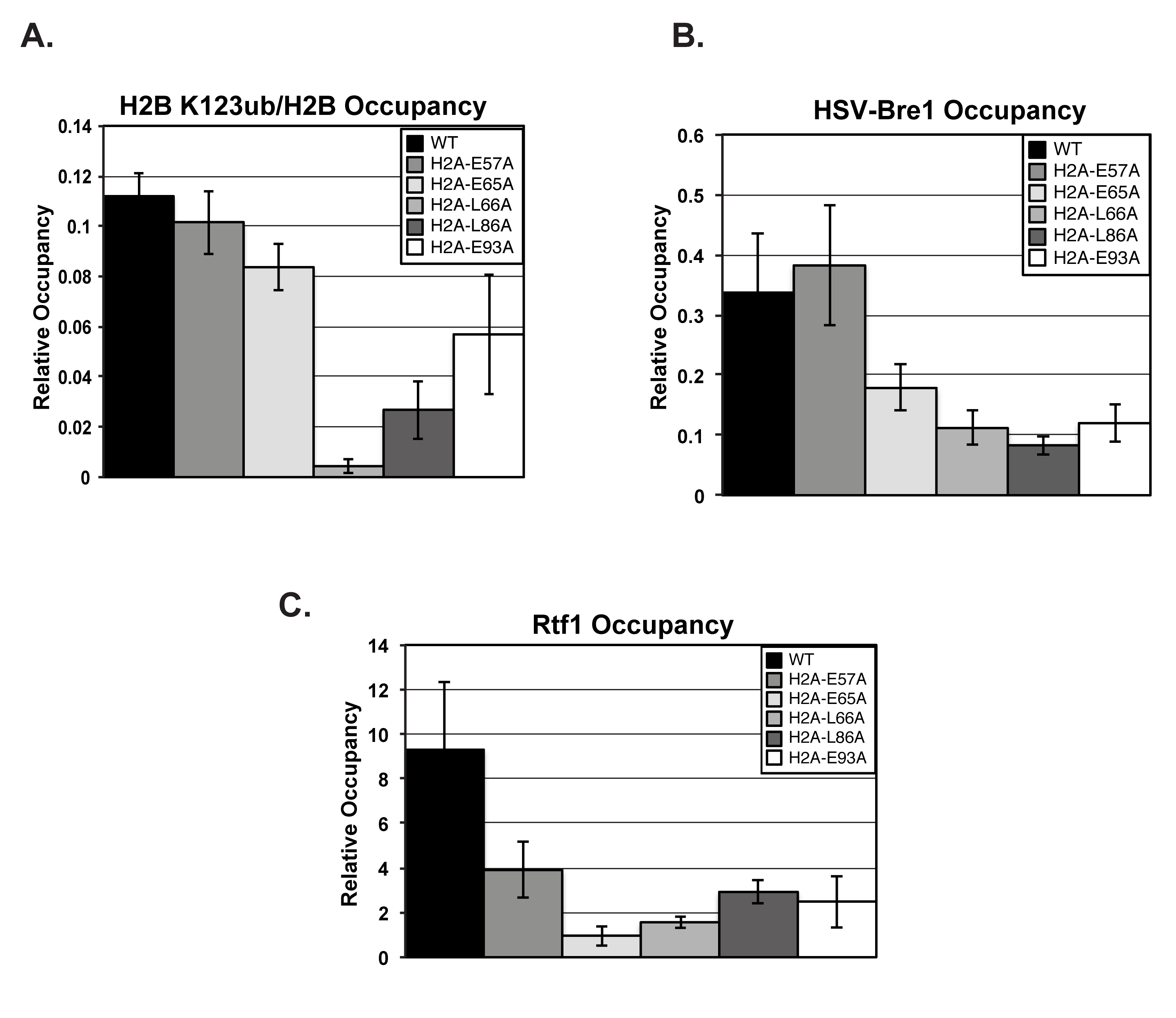

Supplement: S2 Fig — ChIP analysis of H2B K123ub (A), HSV-Bre1 (B), and Rtf1 (C) occupancy at the ADH1 ORF. The error bars represent SEM of three independent experiments. (TIF) [file pgen.1005420.s002.tif]

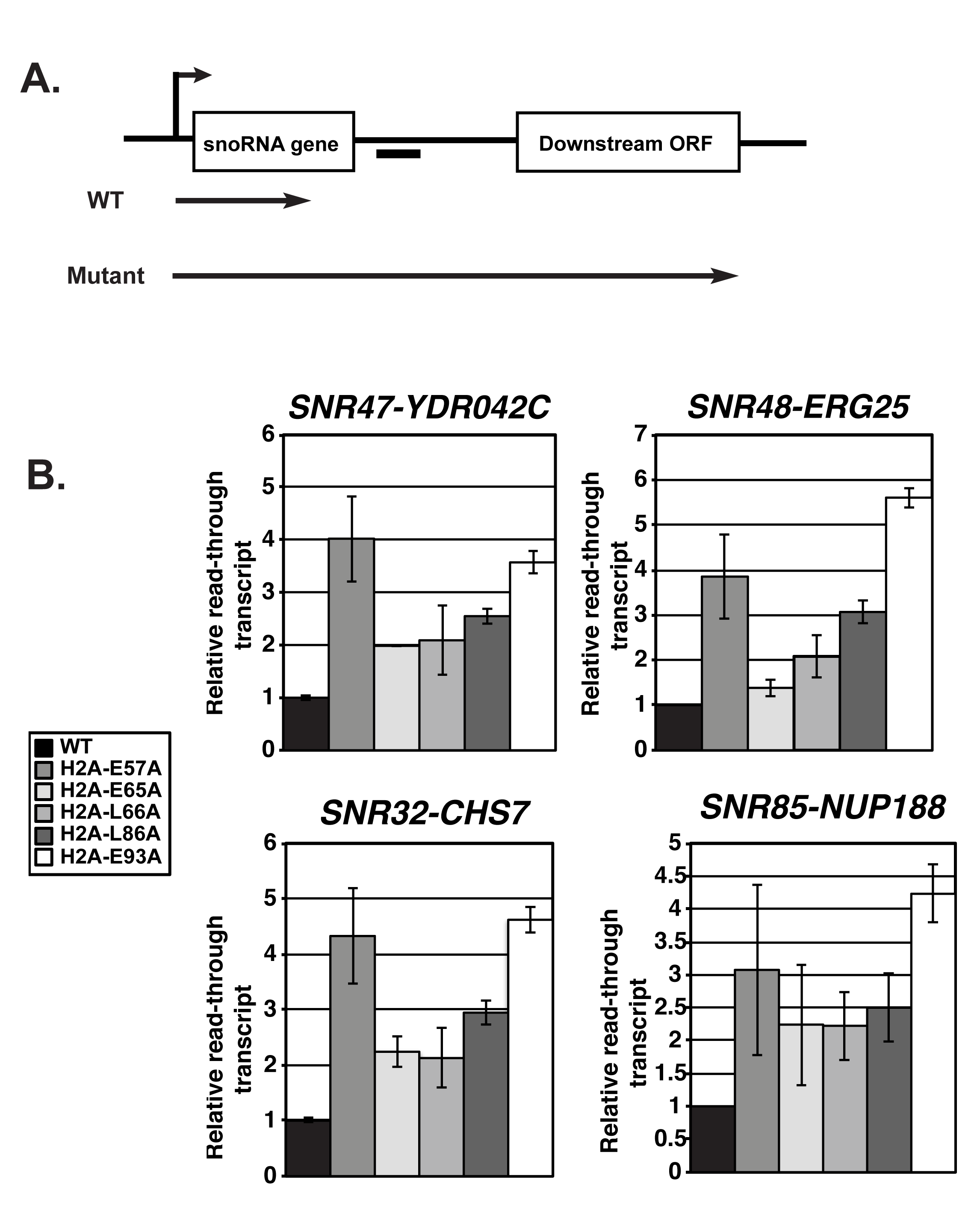

Supplement: S3 Fig — (A) Diagram of a snoRNA gene and the location of qPCR primers used to assess read-through transcription. (B) RT-qPCR analysis of RNA levels downstream of four different snoRNA genes in the H2A mutant strains. Transcript levels in the wild-type control strain were set to 1 and error bars represent SEM of three biological replicates. (TIF) [file pgen.1005420.s003.tif]

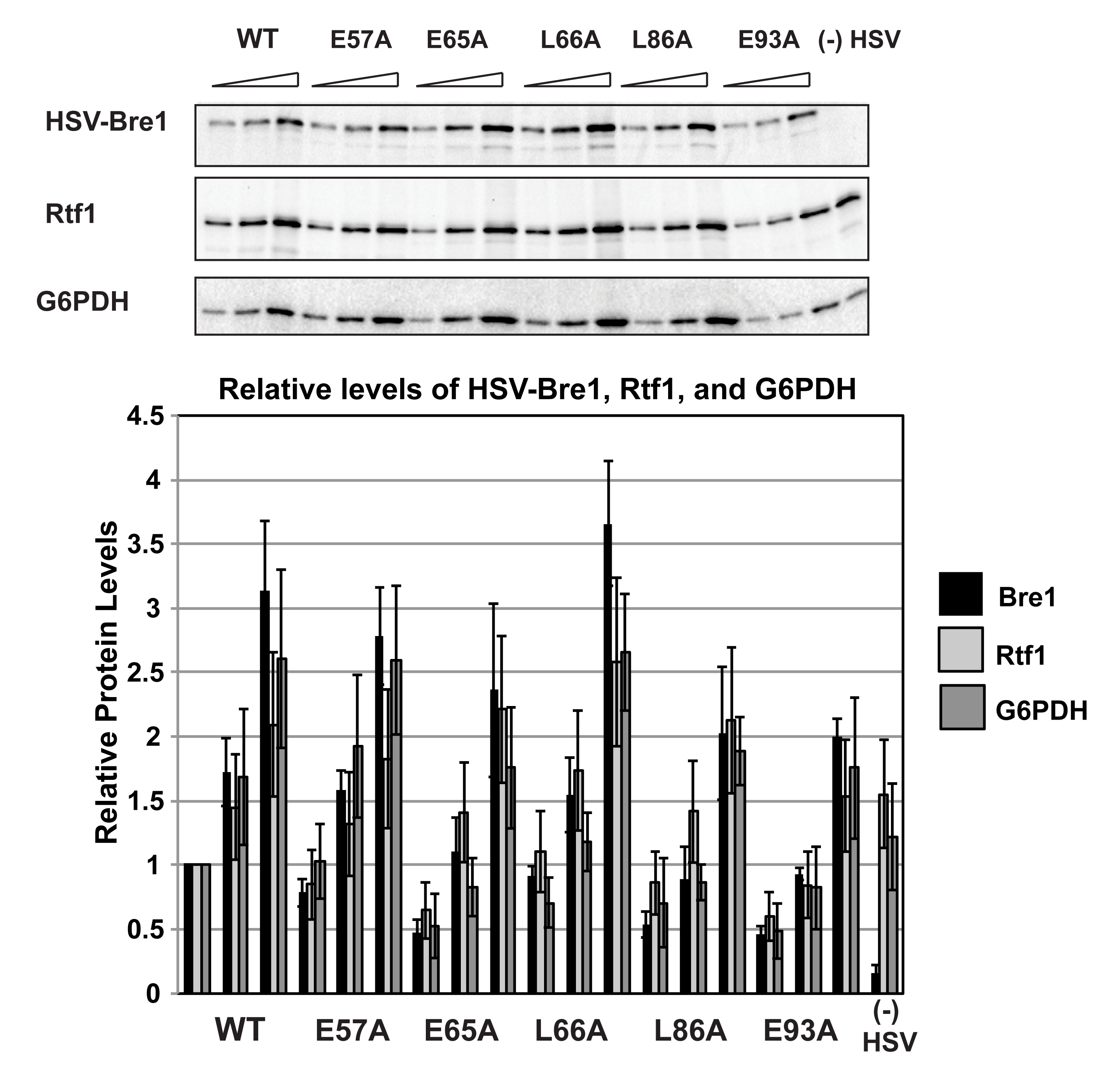

Supplement: S4 Fig — 1-fold, 1.5-fold, and 2-fold concentrations of protein extracts were loaded on SDS polyacrylamide gels and analyzed by western blotting using anti-HSV, anti-Rtf1, and anti-G6PDH, as a loading control. Values were normalized to the initial wild-type protein concentration. Mutant strains were transformants of KY2674. (TIF) [file pgen.1005420.s004.tif]

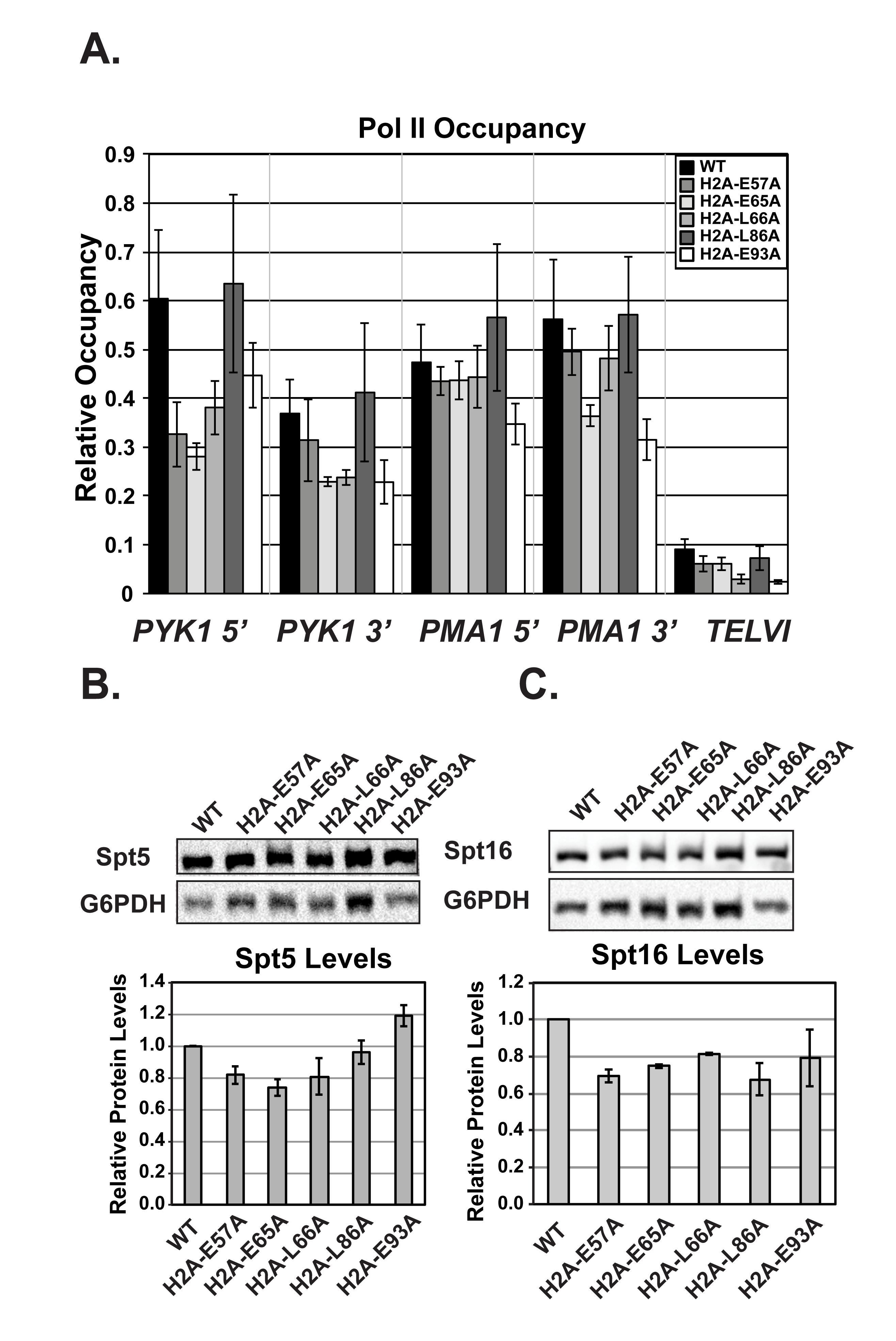

Supplement: S5 Fig — (A) ChIP analysis of Pol II (KY943) at the 5’- and 3’-ends of transcribed loci (PYK1 and PMA1) and at TELVI. The error bars represent SEM of three independent experiments. Western analyses of Spt5 (B), and Spt16 (C) to measure total protein levels in the H2A mutant cells. Values represent protein levels normalized to G6PDH with the wild-type ratio set to one. (TIF) [file pgen.1005420.s005.tif]

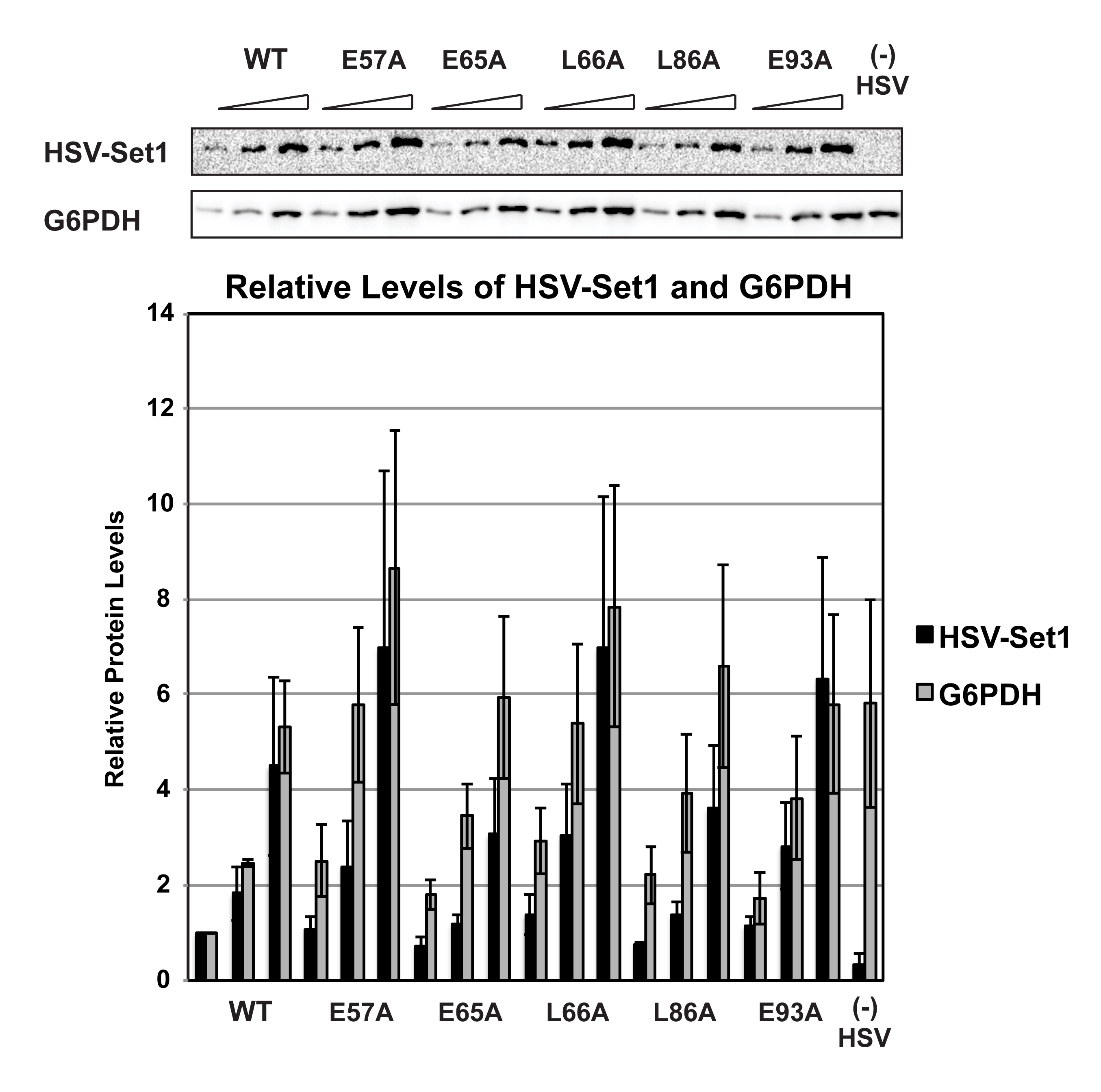

Supplement: S6 Fig — 1-fold, 1.5-fold, and 2-fold concentrations of protein extracts were loaded on SDS polyacrylamide gels and analyzed by western blotting using anti-HSV and anti-G6PDH, as a loading control. Values were normalized to the initial wild-type protein concentration. (TIF) [file pgen.1005420.s006.tif]

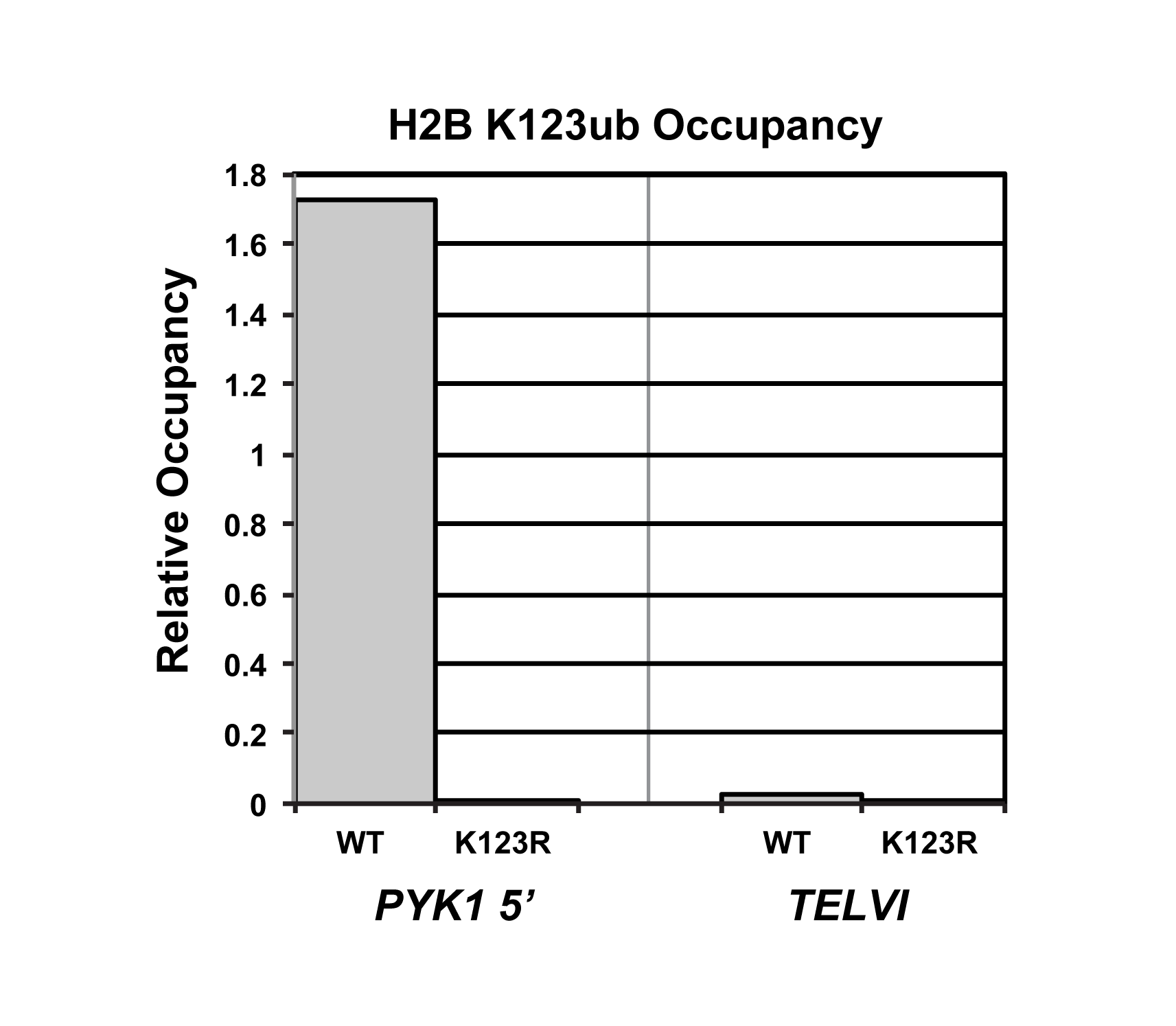

Supplement: S7 Fig — ChIP analysis of H2B K123ub occupancy at the 5'-end of PYK1 and at a nontranscribed region, TELVI. The TELVI locus served as a negative control for wild-type chromatin and the K123R strain served as a negative control for the ChIP experiment. (TIF) [file pgen.1005420.s007.tif]
