# Supplementary material for: The Nucleosome Acidic Patch Regulates the H2B K123 Monoubiquitylation Cascade and Transcription Elongation in Saccharomyces cerevisiae
Source: PLoS Genet. 2015 Aug 4;11(8):e1005420. doi: 10.1371/journal.pgen.1005420 (PMC4524731; doi:10.1371/journal.pgen.1005420)
Supplement: S1 Table — (DOCX) [file pgen.1005420.s008.docx]

**S1 Table. Yeast strains used in this study**

| **Strain** | ***MAT*** | **Genotype** |
| --- | --- | --- |
| KY943 | **a** | (*hta1-htb1*)Δ::*LEU2* (*hta2-htb2)Δ::TRP1 his3Δ200 lys2-128δ leu2Δ1 ura3-52* [pSAB6 = *URA3/C/A/HTA1-HTB1*] |
| KY981 | **a** | (*hta1-htb1*)Δ::*LEU2* *hta2-htb2Δ::TRP1 rkr1∆KanMX4 his3Δ200 lys2-128δ leu2Δ1 ura3-52* [pSAB6 = *URA3/C/A/HTA1-HTB1*] |
| KY1599 | **a** | *(hta1-htb1)∆::LEU2 (hta2-htb2)∆::KanMX6 rtf1∆KanMX4 his3∆200 lys2-128δ leu2*∆*1 trp1∆63 ura3-52* [pSAB6 = *URA3/C/A/HTA1-HTB1*] |
| KY1700 | α | *paf1∆::KanMX4* |
| KY1715 | **a** | *set1∆::KanMX4* |
| KY1717 | **a** | *dot1∆::KanMX4* |
| KY1716 | **a** | *set2∆::KanMX4* |
| KY2044 | **a** | *HTA1-htb1K123R (hta2-htb2*)∆::*KanMX his3*∆*200 leu2*∆*1 trp1*∆*63 ura3*∆*0* |
| KY2086 | α | *(hta1-htb1)∆::LEU2 (hta2-htb2)∆::KanMX ubp8∆::NATMX his3∆200* *lys2-128δ leu2∆1 trp1∆63 ura3-52* [pDC92 = *URA3/HTA1-HTB1*/2-micron] |
| KY2249 | **a** | (*hta1-htb1*)Δ::*LEU2* (*hta2-htb2)Δ::TRP1 his3Δ200 lys2-128δ leu2Δ1 ura3-52* [pAY01 = *HIS3/C/A/HTA1-FLAG-HTB1*] |
| KY2265 | **a** | *(hta1-htb1)∆::LEU2 (hta2-htb2)∆::TRP1 his3∆200 lys2-128δ leu2∆1 trp1∆63 ura3-52* [pRS313 = *HIS3*/C/A] [pSAB6 = *URA3/C/A/HTA1-HTB1*] |
| KY2674 | **a** | *(hta1-htb1)∆::LEU2 (hta2-htb2)∆::TRP1 3XHSV-BRE1 his3∆200 leu2∆1 trp1∆63 ura3-52* [pSAB6 = *URA3/C/A/HTA1-HTB1*] |
| KY2675 | α | *(hta1-htb1)∆::LEU2 (hta2-htb2)∆::KanMX6, RAD6-13XMYC::KanMX his3∆200 leu2∆1 trp1∆63 ura3-52* [pSAB6 = *URA3/C/A/HTA1-HTB1*] |
| KY2676 | **a** | (*hta1-htb1*)Δ::*LEU2* (*hta2-htb2Δ)::TRP1 GAL1pr-YLR454W::KanMX6 his3Δ200 lys2-128δ leu2Δ1 ura3-52* [pSAB6 = *URA3/C/A/HTA1-HTB1*] |
| KY2677 | α | wild-type prototroph |
| KY2678 | α | *spt6-1004* |
| KY2679 | α | *spt16-197* |
| KY2719 | **a** | *(hta1-htb1)∆::LEU2 (hta2-htb2)∆::KanMX6 3XHSV-SET1 his3∆200 leu2*∆*1 trp1∆63 ura3-52 arg4-12* [pSAB6 = *URA3/C/A/HTA1-HTB1*] |
