# Supplementary material for: The Nucleosome Acidic Patch Regulates the H2B K123 Monoubiquitylation Cascade and Transcription Elongation in Saccharomyces cerevisiae
Source: PLoS Genet. 2015 Aug 4;11(8):e1005420. doi: 10.1371/journal.pgen.1005420 (PMC4524731; doi:10.1371/journal.pgen.1005420)
Supplement: S2 Table — (DOCX) [file pgen.1005420.s009.docx]

**S2 Table. Oligonucleotide primers used in this study**

| **Primer** | **Direction** | **Sequence** | **Reference** |
| --- | --- | --- | --- |
| Removal of the FLAG tag | F  R | 5'-CCATACACACATACAATGTCTGCTAAAGCCG-3'  5'-CGGCTTTAGCAGACATTGTATGTGTGTATGG-3' | This study |
| *PYK1* 5' ChIP primer set | F  R | 5'ACGATCTTCTACAATATCGATTCTACCA-3'  5'-TTCTTACGAATACCACAAGTCTGTCA-3' | [1] |
| *PYK1* 3' ChIP primer set | F  R | 5'-GCAATGGCCAATGGTCTACCT-3'  5'-AACCTCCACCACCGAAACC-3' | [1] |
| *PMA1* 5' ChIP primer set | F  R | 5'-GCTAGACCAGTTCCAGAAGAATATTTACA-3'  5'-CAGCCATTTGATTCAAACCGTA-3 | [1] |
| *PMA1* 3' ChIP primer set | F  R | 5'-GAAATCTTCTTGGGTCTATGGATTG-3'  5'-CAACATCAGCGAAAATAGCGAT-3' | [1] |
| *TELVI* ChIP primer set | F  R | 5'-TGCAAGCGTAACAAAGCCATA-3'  5'-TCCGAACGCTATTCCAGAAAG-3' | [1] |
| *SER3* Northern probe | F  R | 5'-TCTGCTAAGATCTCAATTAGATTG-3'  5'-CAAGGATGTCATCGAAGAGGC-3' | [2] |
| *SRG1* Northern probe | F  R | 5'-TGGTTAAGCAGTTAGGCTGG-3'  5'-TTTCCTTATCCTCTGCTCCC-3' | [2] |
| *SCR1* Northern probe | F  R | 5'-CAACTTAGCCAGGACATCCA-3'  5'-AGAGAGACGGATTCCTCACG-3' | [2] |
| *FLO8* Northern probe | F  R | 5'-TGATGCCACTAAGGATGAGA-3'  5'-GGTCTTCAACCATACCAATA-3' | [2] |
| *SNR47-YDR042C* qRT-PCR | F  R | 5'-CAACAACATGAATTTCTTCGTCCGAATCC-3'  5'-CCGCCTTTCTTCTTGGAAATTGGTAACAGG-3' | [3] |

**References for S2 Table**

1. Liu Y, Warfield L, Zhang C, Luo J, Allen J, et al. (2009) Phosphorylation of the transcription elongation factor Spt5 by yeast Bur1 kinase stimulates recruitment of the PAF complex. Mol Cell Biol 29: 4852-4863.

2. Hainer SJ, Martens JA (2011) Identification of histone mutants that are defective for transcription-coupled nucleosome occupancy. Mol Cell Biol 31: 3557-3568.

3. Tomson BN, Crisucci EM, Heisler LE, Gebbia M, Nislow C, et al. (2013) Effects of the Paf1 complex and histone modifications on snoRNA 3'-end formation reveal broad and locus-specific regulation. Mol Cell Biol 33: 170-182.
