# Supplementary material for: The Nucleosome Acidic Patch Regulates the H2B K123 Monoubiquitylation Cascade and Transcription Elongation in Saccharomyces cerevisiae
Source: PLoS Genet. 2015 Aug 4;11(8):e1005420. doi: 10.1371/journal.pgen.1005420 (PMC4524731; doi:10.1371/journal.pgen.1005420)
Supplement: S3 Table — (DOCX) [file pgen.1005420.s010.docx]

**S3 Table. Plasmids used in this study**

| **Plasmid** | **Histone** | **Derivation and reference** |
| --- | --- | --- |
| pAY01 | WT untagged | Site directed mutagenesis of pZS145; this study |
| pCEC01 | F26A | This study. Site directed mutagenesis of library plasmid from [1] |
| pCEC02 | E57A | This study. Site directed mutagenesis of library plasmid from [1] |
| pCEC03 | E65A | This study. Site directed mutagenesis of library plasmid from [1] |
| pCEC04 | L66A | This study. Site directed mutagenesis of library plasmid from [1]. |
| pCEC05 | L86A | This study. Site directed mutagenesis of library plasmid from [1] |
| pCEC06 | E93A | This study. Site directed mutagenesis of library plasmid from [1] |
| pCEC07 | L94A | This study. Site directed mutagenesis of library plasmid from [1] |
| pCEC08 | H113A | This study. Site directed mutagenesis of library plasmid from [1] |
| pJH23KR | K123R | Site directed mutagenesis of pJH23 [2] |
| pZS145 | WT FLAG-H2B | [3] |

**References for S3 Table**

1. Nakanishi S, Sanderson BW, Delventhal KM, Bradford WD, Staehling-Hampton K, et al. (2008) A comprehensive library of histone mutants identifies nucleosomal residues required for H3K4 methylation. Nat Struct Mol Biol 15: 881-888.

2. Ng HH, Xu RM, Zhang Y, Struhl K (2002) Ubiquitination of histone H2B by Rad6 is required for efficient Dot1-mediated methylation of histone H3 lysine 79. J Biol Chem 277: 34655-34657.

3. Sun ZW, Allis CD (2002) Ubiquitination of histone H2B regulates H3 methylation and gene silencing in yeast. Nature 418: 104-108.
